# Circulating T cell atlas in Moyamoya disease: insights into immunopathogenesis of cerebrovascular disorders

**DOI:** 10.1186/s12974-025-03479-3

**Published:** 2025-06-05

**Authors:** Chenglong Liu, Junsheng Li, Siqi Mou, Yuheng Pang, Liujia Chan, Qiheng He, Wei Liu, Zhikang Zhao, Bojian Zhang, Zhiyao Zheng, Wei Sun, Xiangjun Shi, Qian Zhang, Rong Wang, Yan Zhang, Wenjing Wang, Dong Zhang, Peicong Ge

**Affiliations:** 1 Department of Neurosurgery, Beijing Tiantan Hospital, Capital Medical University, Beijing, 100070 China; 2 China National Clinical Research Center for Neurological Diseases, Beijing, 100070 China; 3 Medical School, University of Chinese Academy of Sciences, Beijing, 101408 China; 4 Beijing YouAn Hospital, Beijing Institute of Hepatology, Capital Medical University, Beijing, 100069 China; 5 Department of Medicinal Chemistry, College of Pharmaceutical Sciences, Capital Medical University, Beijing, 100069 P. R. China; 6 Department of Neurosurgery, Beijing Hospital, National Center of Gerontology, Beijing, 100730 China; 7 Institute of Geriatric Medicine, Chinese Academy of Medical Sciences, Beijing, 100730 China

**Keywords:** Moyamoya disease, T cells, Mitochondrial dysfunction, Immunometabolism, Mass cytometry

## Abstract

**Background:**

Moyamoya disease (MMD) is a chronic cerebrovascular disorder characterized by progressive stenosis or occlusion of the intracranial arteries, accompanied by the formation of fragile collateral vessels, ultimately leading to ischemic or hemorrhagic strokes. Immune dysregulation, particularly involving T cell abnormalities and mitochondrial dysfunction, plays a critical role in the pathogenesis of MMD; however, their precise relationship remains unclear.

**Methods:**

Peripheral blood mononuclear cells (PBMCs) from patients with MMD and healthy controls were analyzed using mass cytometry (CyTOF) and transcriptomic profiling. Additionally, clinical characteristics and neuroimaging data were collected to perform integrated correlation analyses with immune profiling data.

**Results:**

Patients with MMD exhibited aberrant T cell activation and altered subset distribution, accompanied by mitochondrial dysfunction and impaired oxidative phosphorylation capacity. Increased oxidative stress and endoplasmic reticulum stress were observed in T cells, along with disease-specific downregulation of immune checkpoint molecules, including PD-1 and ICOS.

**Conclusions:**

This study highlights the critical involvement of immune activation and mitochondrial dysfunction in the pathophysiology of MMD, providing novel insights into disease mechanisms and identifying immunometabolic pathways as potential targets for therapeutic intervention.

**Supplementary Information:**

The online version contains supplementary material available at 10.1186/s12974-025-03479-3.

## Introduction

Moyamoya disease (MMD) represents a significant clinical and research challenge within the spectrum of cerebrovascular disorders. It is characterized by progressive stenosis or occlusion at the terminal portions of the internal carotid arteries, accompanied by the formation of abnormal collateral vessels known as “moyamoya” vessels [1]. MMD is one of the most prevalent pediatric cerebrovascular conditions in East Asia, with a steadily increasing incidence [2, 3]. Clinically, it typically presents with transient ischemic attacks and ischemic strokes due to compromised major arteries, as well as hemorrhagic strokes caused by the rupture of fragile collateral vessels or associated aneurysms [4, 5]. Although the underlying pathogenesis remains incompletely understood, emerging evidence suggests that immune dysregulation is closely associated with both the onset and progression of MMD, indicating a bidirectional interplay between immune dysregulation and disease development [6–9]. 

In the peripheral immune environment, peripheral blood mononuclear cells (PBMCs) play a pivotal role, with T cell subpopulations—highly abundant and phenotypically diverse—undergoing dynamic alterations across a variety of disease states [10–12]. These changes involve not only shifts in subset proportions but also functional reprogramming of T cells [13, 14]. In MMD, previous studies have reported an imbalance between T helper 17 (Th17) cells and regulatory T (Treg) cells in peripheral blood, while early autopsy analyses have revealed infiltration of T cells and macrophages in the stenotic vessel walls [15, 16]. Our prior work using single-cell RNA sequencing of peripheral blood samples from MMD patients identified a reduction in effector T cells and an expansion of Treg cells, accompanied by inflammatory activation of innate immune cells within PBMCs [17, 18]. Multiple factors may contribute to peripheral immune dysregulation. We hypothesize that chronic cerebral ischemia and hypoxia, secondary to intracranial arterial stenosis or occlusion in MMD, may act in concert with sustained antigenic stimulation to drive maladaptive alterations in circulating immune cell populations.

Cellular metabolism is fundamental to immune cell function, supporting environment-dependent processes such as migration, proliferation, and effector molecule secretion through dynamic metabolic reprogramming [19]. Mitochondria serve as central regulators of both metabolism and intracellular signaling, playing an essential role in cell survival; their dysfunction has been implicated in a wide range of pathological conditions [20]. In MMD, transcriptomic analyses of vascular tissues have revealed downregulation of genes involved in mitochondrial function and oxidative phosphorylation pathways [21]. Moreover, mitochondrial dysfunction has been reported in circulating endothelial colony-forming cells derived from patients with MMD. Notably, homozygous variants in *TOMM7* (translocase of the outer mitochondrial membrane 7) have been associated with microcephalic osteodysplastic primordial dwarfism syndromes that co-occur with MMD [22, 23]. While the relationship between MMD and mitochondrial dysfunction may be bidirectional, a critical knowledge gap remains regarding the metabolic heterogeneity of circulating immune cell subsets—particularly T cells—and their contribution to MMD pathophysiology.

Although previous studies have demonstrated that distinct metabolic features influence T cell differentiation and function, the relationship between mitochondrial activity, metabolism-related signaling pathways, and peripheral T cell subsets in MMD remains poorly characterized. To address this gap, the present study employs mass cytometry (CyTOF) to perform a comprehensive immunophenotypic analysis of peripheral T cells in patients with MMD, aiming to delineate the T cell landscape and its subset-specific alterations. In particular, characterizing mitochondrial function and immunometabolic states under disease conditions may uncover novel therapeutic targets and inform personalized clinical strategies. Beyond MMD, this integrative approach may provide broader insights into the immunopathology of other chronic ischemic and immune-mediated vascular diseases.

## Methods

### Participants and sample collection

Patients diagnosed with MMD were recruited from Beijing Tiantan Hospital. All patients met the 2021 Japanese diagnostic criteria, which require evidence of stenosis or occlusion at the terminal portion of the internal carotid artery and the proximal segments of the middle and anterior cerebral arteries [24]. Individuals with autoimmune diseases were excluded based on clinical history and laboratory results. Age- and sex-matched healthy controls (HCs) were enrolled, with exclusion criteria including chronic illnesses and the use of immunomodulatory medications. Following an overnight fast (≥ 8 h), peripheral blood samples were collected into EDTA-coated anticoagulant tubes, maintained under cold conditions, and processed within two hours of collection. PBMCs were isolated using Ficoll-Paque density gradient centrifugation (500 × g, 10 min), resuspended in cryopreservation medium containing 10% dimethyl sulfoxide (DMSO) and 90% fetal bovine serum (FBS), and subjected to controlled-rate freezing prior to storage in liquid nitrogen for subsequent experiments.

### Data collection

Baseline clinical characteristics of patients with MMD were recorded at the time of admission, including age, sex, medical history (hypertension, diabetes mellitus, and dyslipidemia as defined by international guidelines), smoking and alcohol consumption status, and initial clinical presentation (ischemic or hemorrhagic). Basic demographic and health information for the HC group was obtained through structured interviews and verified using health examination records. Imaging evaluation of MMD patients was conducted based on the Suzuki staging system [25]. Digital subtraction angiography (DSA) images were independently reviewed in a blinded manner by two experienced neurosurgeons who were unaware of all other patient information. In cases of disagreement, a third expert was consulted to reach consensus. The Suzuki stage was determined according to the hemisphere with the most severe lesion, and the contralateral hemisphere was also assessed for reference.

### Mass cytometry experimental procedures

Monoclonal antibody selection was guided by the objective of profiling immune cells and T cell subsets, based on prior studies of the single-cell metabolic landscape of human cytotoxic T cells (see Table S1) [26]. Cryopreserved PBMCs were thawed, resuspended, and washed in phosphate-buffered saline (PBS) supplemented with 2% FBS. Cell viability was assessed using cisplatin staining, following the manufacturer′s protocol. Surface markers were labeled using metal isotope-conjugated monoclonal antibodies, followed by intracellular staining. After staining, cells were washed twice and incubated with a DNA intercalator solution for nuclear labeling. Data acquisition was performed using a Helios mass cytometer (Fluidigm, USA).

### Mass cytometry data processing and analysis

Raw mass cytometry data were initially preprocessed through manual gating to exclude dead cells, isolate single live cells, and identify CD45⁺ immune cell populations for downstream analysis. Signal intensities were arcsinh-transformed to improve clustering accuracy and reduce data skewness. High-dimensional data analysis was performed using the PhenoGraph algorithm implemented in the R package Cytofkit (version 1.10.0) [27]. Cluster frequencies, heterogeneity, and molecular expression patterns were visualized using t-distributed stochastic neighbor embedding (t-SNE). Phenotypically similar clusters were manually merged to reduce redundancy. Cell counts and marker expression values were then extracted for each cluster. To facilitate intergroup comparisons, molecular expression data were normalized. All statistical analyses and visualizations were conducted using the R packages pheatmap (version 1.0.12) and ggplot2 (version 3.4.0).

### RNA-seq library preparation and data analysis

PBMC samples were obtained from 13 patients with MMD and 6 HCs for RNA sequencing (RNA-seq). Total RNA was extracted and used to construct sequencing libraries, which were subjected to paired-end sequencing on the Illumina NovaSeq 6000 and HiSeq platforms, generating 150 bp (bp) paired-end reads. Data preprocessing was performed using the data.table package in R. Genes with a maximum expression value below 10 were excluded. For duplicated genes, only the transcript with the highest average expression was retained. Additionally, genes expressed in fewer than 50% of the samples were filtered out to ensure consistency in the analyzed gene set. Gene expression levels were quantified as counts per million (CPM) using edgeR package, and normalization was performed using limma [28, 29]. Principal component analysis (PCA) was conducted on the normalized expression matrix to assess sample distribution, using the HC group as a reference. Differential gene expression analysis was performed using DESeq2 [30]. Genes with an absolute fold change > 1.5 and an adjusted *p*-value < 0.05 were considered differentially expressed. Gene Ontology (GO) and Kyoto Encyclopedia of Genes and Genomes (KEGG) pathway enrichment analyses were conducted using the clusterProfiler package to elucidate the biological processes and signaling pathways associated with these genes [31]. 

### Single-cell RNA-seq data processing workflow

Raw gene expression matrices for the HC group were obtained from the GEO database (accession number: GSE165080) [32], while datasets for the MMD group were sourced from the China National Genomics Data Center (GSA-Human, accession number: HRA005203) [17]. Control samples were age- and sex-matched to ensure cohort comparability. During quality control, genes expressed in fewer than three cells, cells with fewer than 500 detected genes, or cells exhibiting mitochondrial gene proportions exceeding 20% or red blood cell (RBC) gene proportions surpassing 5% were excluded. The filtered and merged dataset was normalized using Seurat′s `NormalizeData` function, with the top 2000 highly variable genes identified using the `FindVariableFeatures` function. Batch effects were corrected using the Harmony package. Differentially expressed genes (DEGs) were identified using Seurat′s `FindMarkers` function with the Wilcoxon rank-sum test, applying a threshold of|log₂(fold change)| ≥ 1 and a false discovery rate (FDR) ≤ 0.05, as calculated using the Benjamini–Hochberg method. Functional enrichment analysis was subsequently performed on the DEGs to elucidate their biological relevance and associated signaling pathways.

### Statistical analysis

Statistical analyses were performed using R software (version 4.3.1). Continuous variables were presented as mean ± standard deviation (SD), and categorical variables were summarized as counts and percentages. Group comparisons of continuous variables were conducted using the Wilcoxon rank-sum test (also known as the Mann–Whitney U test), while categorical variables were compared using the χ² test or Fisher’s exact test, as appropriate. Pearson’s correlation analysis was used to evaluate relationships between continuous variables. Spearman correlation analysis was performed to evaluate relationships between immune cell proportions, molecular expression levels, and Suzuki stages. A two-sided *p*-value < 0.05 was considered statistically significant.

## Results

### Immune and metabolic alterations in peripheral blood T cells of MMD patients

**Fig. 1:**
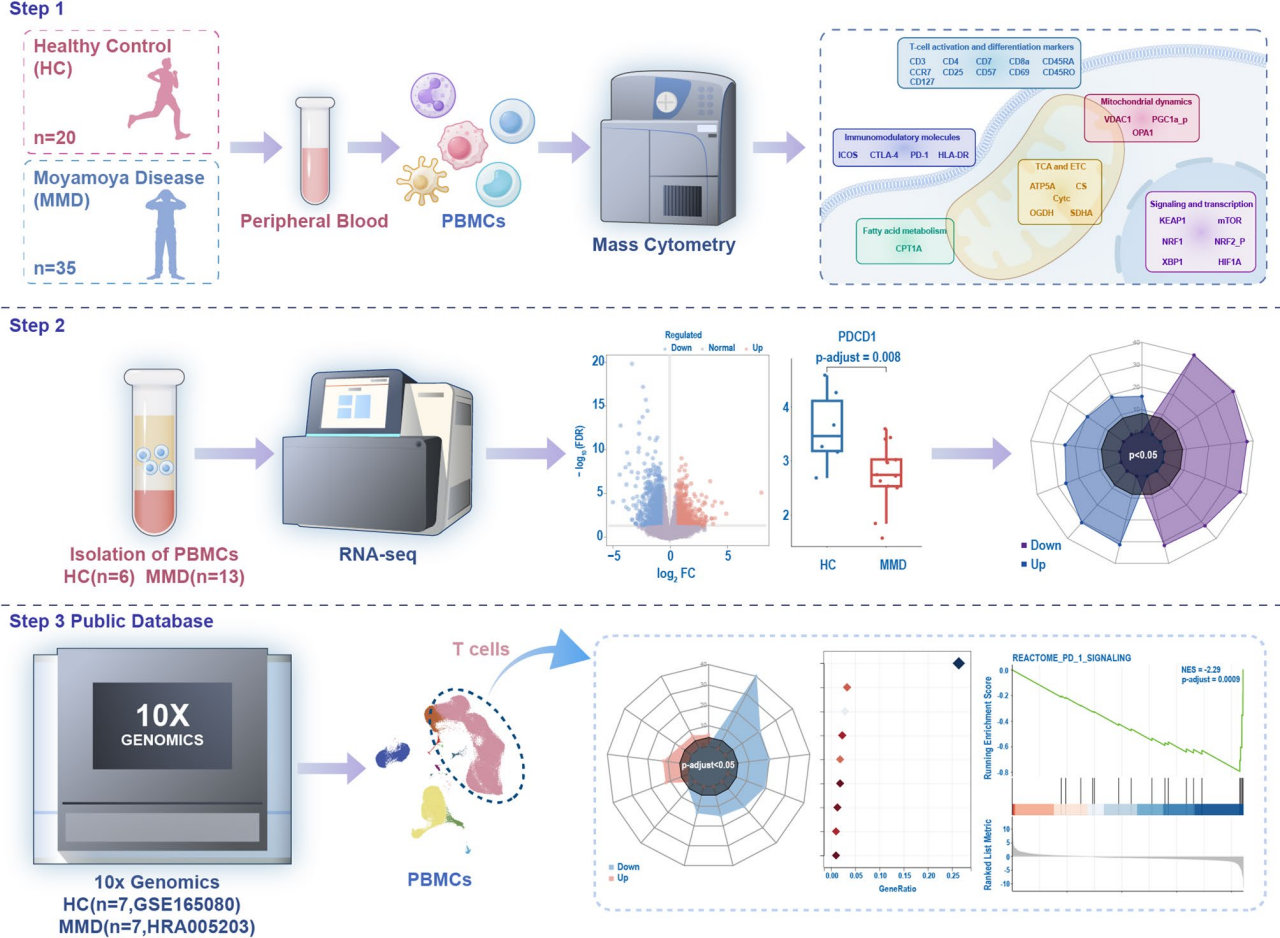
Experimental design. Abbreviations: ATP5A, ATP synthase subunit alpha, mitochondrial; CCR7, C-C chemokine receptor type 7; CPT1A, carnitine palmitoyltransferase 1A; CS, citrate synthase; CTLA-4, cytotoxic T-lymphocyte protein 4; CytC, cytochrome c; HC, healthy control; HIF1A, hypoxia-inducible factor 1-alpha; HLA-DR, human leukocyte antigen–DR isotype; ICOS, inducible T-cell costimulator; IDH, isocitrate dehydrogenase; KEAP1, Kelch-like ECH-associated protein 1; MMD, moyamoya disease; mTOR, mammalian target of rapamycin; NRF1, nuclear respiratory factor 1; NRF2_p, phosphorylated nuclear factor erythroid 2–related factor 2; OGDH, oxoglutarate dehydrogenase; OPA1, optic atrophy 1; PBMCs, peripheral blood mononuclear cells; PD-1, programmed cell death protein 1; PGC1a_p, phosphorylated peroxisome proliferator-activated receptor gamma coactivator 1-alpha; SDHA, succinate dehydrogenase complex flavoprotein subunit A; VDAC1, voltage-dependent anion-selective channel protein 1; XBP1, X-box binding protein 1

**Fig. 2:**
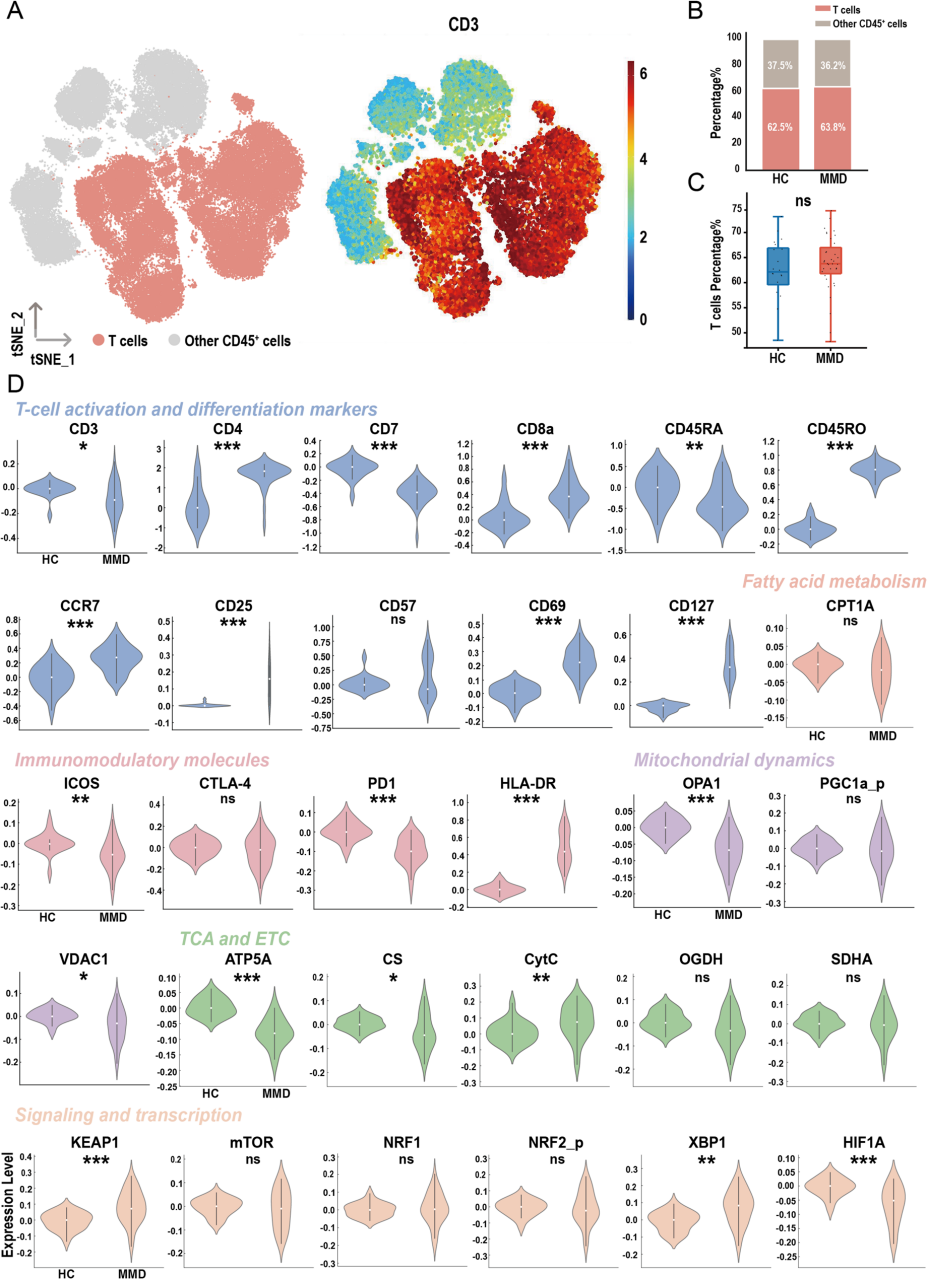
Mass cytometry profiling of the peripheral blood T cell landscape. (**A**) t-SNE plot of T cells and heatmap overlay showing CD3 expression levels. (**B**) Stacked bar chart showing the percentage distribution of T cell subsets. (**C**) Box plot comparing T cell subset proportions between healthy controls (HCs) and patients with moyamoya disease (MMD). (**D**) Comparison of T-cell functional and metabolic marker expression profiles between HC and MMD groups based on mass cytometry analysis. Significance: ns, *p* ≥ 0.05; *p < 0.05; **p < 0.01; ***p < 0.001 (Wilcoxon rank-sum test)

The overall study design is illustrated in Fig. 1. The CyTOF cohort included 20 HCs and 35 patients with MMD, with hypertension significantly more prevalent in the MMD group (Table S2). Dimensionality reduction of peripheral blood CD45⁺ PBMCs was performed to visualize immune cell subsets (Fig. 2A), and T cells were identified based on CD3 expression. Figure 2B shows the distribution of T cells and other CD45⁺ immune cells in both groups, while Fig. 2C demonstrates no significant difference in overall T-cell proportions between them. However, several T-cell activation markers, including CD4, CD8a, CD45RO, CCR7, CD25, CD69, and CD127, were significantly upregulated in the MMD group. Additionally, mitochondrial dynamics and metabolic pathways were altered, as indicated by reduced expression of ATP5A and citrate synthase (CS) in the tricarboxylic acid (TCA) cycle. Notably, cytochrome c (CytC) levels were increased, while OPA1 and VDAC1 expression were significantly decreased, suggesting disrupted mitochondrial function and compromised electron transport chain (ETC) integrity. Furthermore, immunoregulatory molecules PD-1 and ICOS were downregulated, while HLA-DR expression was elevated in MMD patients. Stress-associated signaling molecules, including KEAP1 and XBP1, were elevated, whereas HIF1A was suppressed. Collectively, these findings indicate enhanced T-cell activation, mitochondrial dysfunction, increased oxidative and endoplasmic reticulum stress, and downregulation of immune checkpoint molecules (Fig. 2D).

### Altered T-cell subpopulations in MMD

**Fig. 3:**
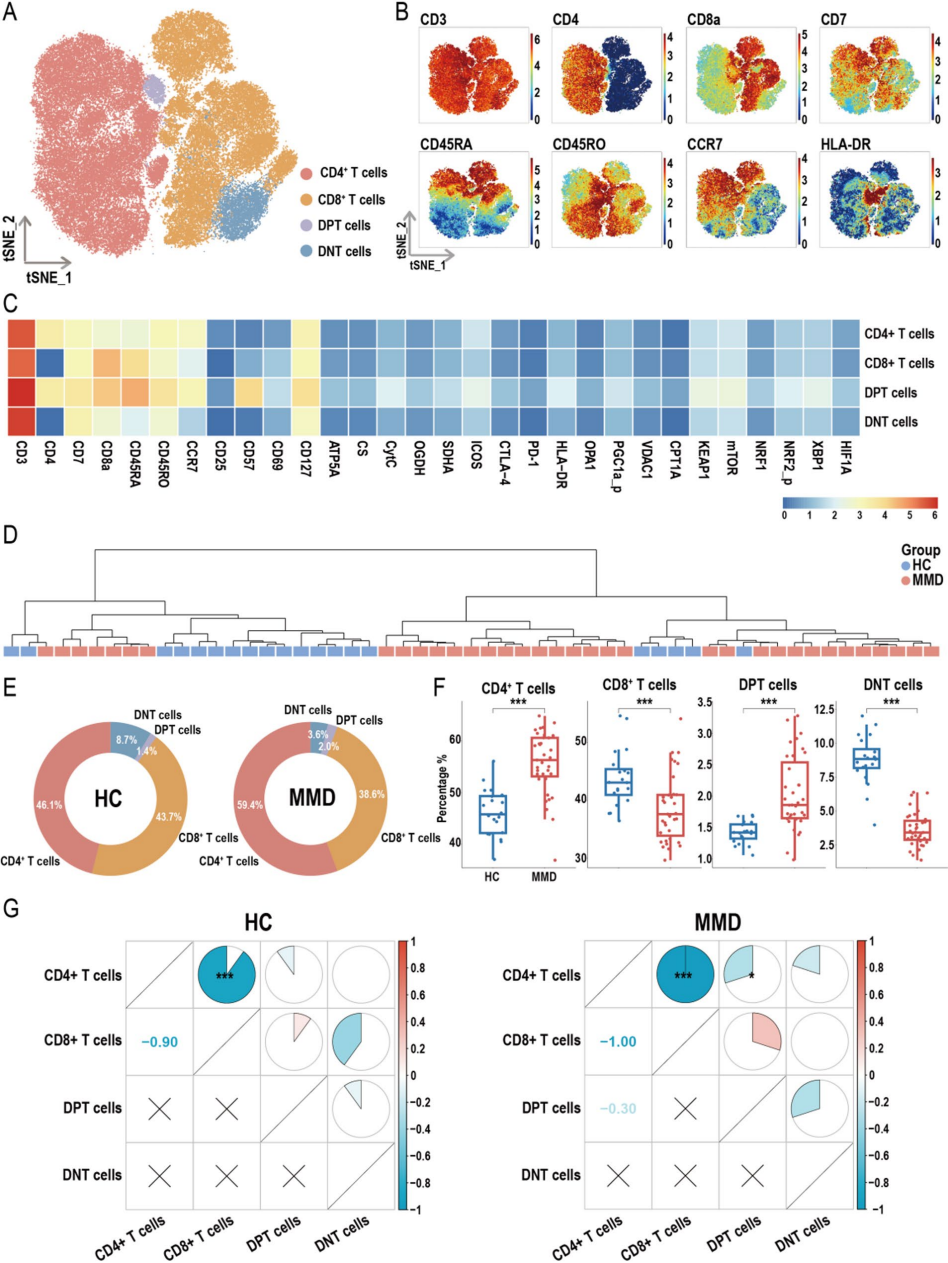
Overview of T cell subsets. (**A**) t-SNE plot showing the distribution of T cell subsets. (**B**) t-SNE heatmap displaying the expression levels of specific marker molecules in T cell subsets. (**C**) Heatmap illustrating the correlation between T cell subsets and their corresponding protein expression profiles from mass cytometry. (**D**) Cluster dendrogram based on the proportions of T cell subsets. (**E**) Circular pie chart showing the proportional distribution of T cell subsets. (**F**) Box plot comparing the proportions of T cell subsets between healthy controls (HCs) and moyamoya disease (MMD) patients (*p*-values were calculated using the Wilcoxon rank-sum test). (**G**) Heatmap showing the intra-group correlations of T cell subset proportions within the HC and MMD groups (*p*-values were calculated using Pearson correlation analysis). Significance: *p < 0.05; ***p < 0.001

Dimensionality reduction of CD3⁺ T cells within the CD45⁺ immune cell population identified four major T-cell subpopulations: CD4⁺ T cells, CD8⁺ T cells, double-positive T cells (DPT), and double-negative T cells (DNT) (Fig. 3A). A t-SNE heatmap of representative clustering markers is shown in Fig. 3B, and the correlations between T-cell subpopulations and protein expression profiles assessed via mass cytometry are presented in Fig. 3C. A dendrogram based on inter-sample correlations of T-cell subset proportions is depicted in Fig. 3D. Figure 3E displays circular bar charts comparing the distributions of T-cell subpopulations between HCs and MMD patients. The analysis revealed significant compositional shifts in T-cell subsets in MMD, characterized by increased proportions of CD4⁺ T and DPT cells, and decreased proportions of CD8⁺ T and DNT cells relative to HCs (Fig. 3F). Notably, a strong inverse correlation was observed between CD4⁺ and CD8⁺ T cells in both groups. In the MMD group specifically, a significant negative correlation was also detected between CD4⁺ T cells and DPT cells (Fig. 3G).

**Fig. 4:**
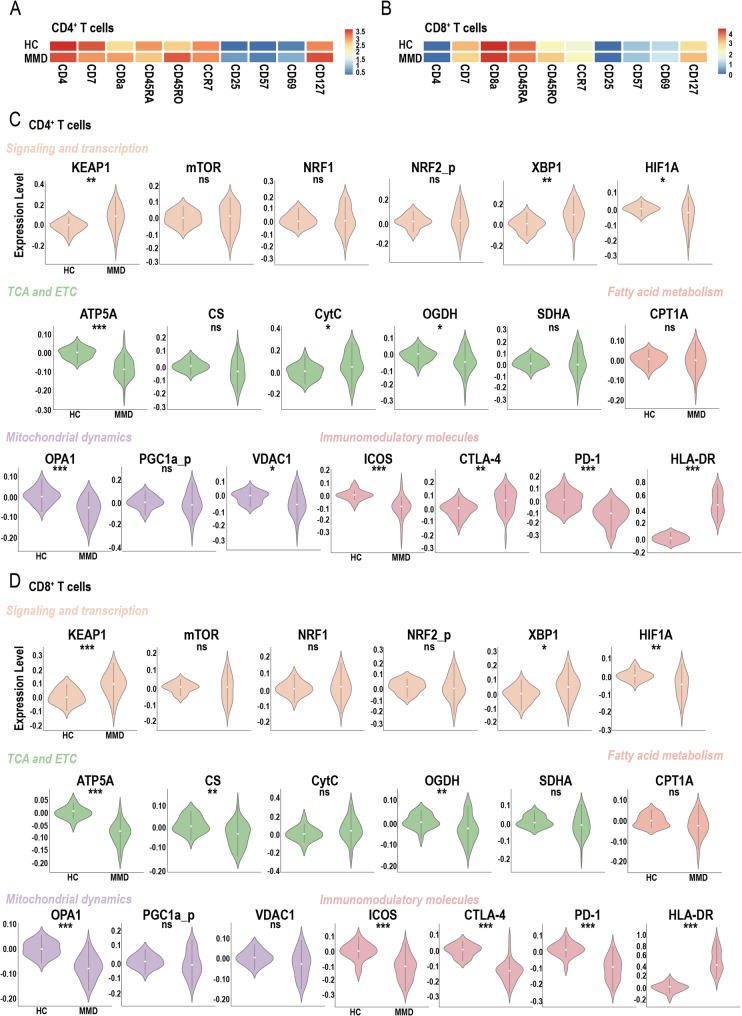
Mass cytometry analysis of molecular expression differences in T cell subsets (CD4 + T and CD8 + T cells) between healthy control (HC) and moyamoya disease (MMD) groups. Heatmap showing the expression of T-cell activation and differentiation markers in (**A**) CD4^+^ T cells and (**B**) CD8^+^ T cells between HC and MMD groups. Differential expression of metabolic and functional state markers analyzed by mass cytometry in (**C**) CD4^+^ T cells and (**D**) CD8^+^ T cells between HC and MMD groups. Significance: ns, *p* ≥ 0.05; *p < 0.05; **p < 0.01; ***p < 0.001 (Wilcoxon rank-sum test)

Heatmaps illustrating protein expression correlations related to T-cell activation and differentiation markers in CD4⁺ and CD8⁺ T cells are shown for both HCs and patients with MMD (Figs. 4A-B). Compared to HCs, the MMD group exhibited markedly elevated expression of CD45RO and CD127, accompanied by reduced CD45RA expression in both CD4⁺ and CD8⁺ T cells. In the CD4⁺ T-cell subset, MMD patients displayed increased levels of KEAP1, XBP1, CytC, HLA-DR, and CTLA-4, along with decreased expression of HIF1A, ATP5A, OGDH, OPA1, VDAC1, PD-1, and ICOS compared to HCs (Fig. 4C). In the CD8⁺ T-cell subset, significantly higher levels of KEAP1, XBP1, and HLA-DR were also observed, while expression of HIF1A, ATP5A, CS, OGDH, OPA1, CTLA-4, PD-1, and ICOS was significantly decreased (Fig. 4D). These results highlight profound alterations in immune activation status and metabolic programming across both CD4⁺ and CD8⁺ T cells in MMD. Additional profiling of DPT and DNT subsets based on mass cytometry analysis is presented in Figure S1.

### Alterations in CD4⁺ and CD8⁺ T-cell subpopulations in MMD

**Fig. 5:**
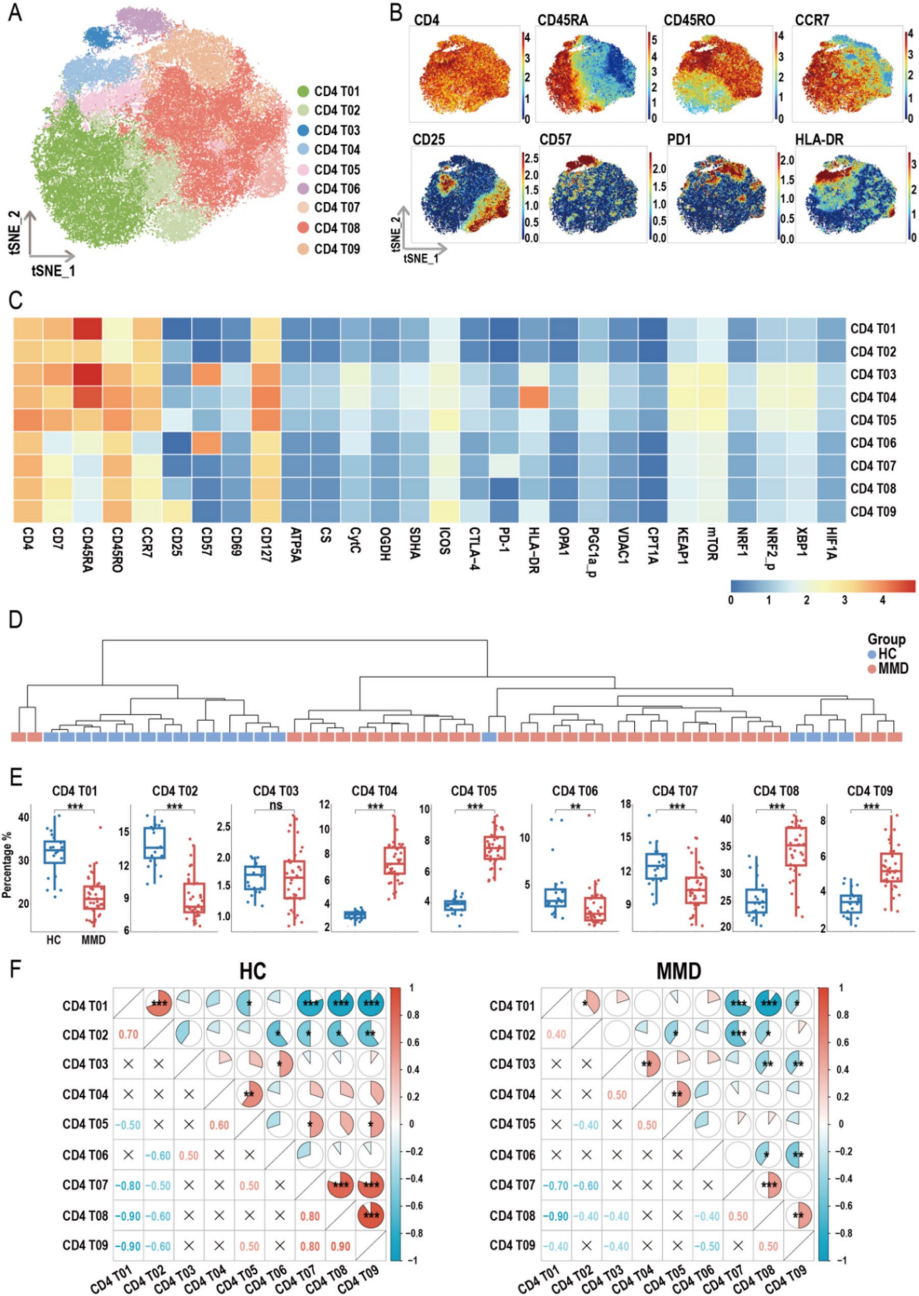
Overview of CD4^+^ T cell subsets. (**A**) t-SNE plot showing the distribution of CD4^+^ T cell subsets. (**B**) t-SNE heatmap displaying the expression levels of specific marker molecules in CD4^+^ T cell subsets. (**C**) Heatmap illustrating the correlation between CD4^+^ T cell subsets and their protein expression profiles measured by mass cytometry. (**D**) Cluster dendrogram based on the proportions of CD4^+^ T cell subsets. (**E**) Box plots comparing the proportions of CD4^+^ T cell subsets between healthy controls (HCs) and moyamoya disease (MMD) patients (*p*-values were calculated using the Wilcoxon rank-sum test). (**F**) Heatmap showing the intra-group correlations of CD4^+^ T cell subset proportions within the HC and MMD groups (*p*-values were calculated using Pearson correlation analysis). Significance: ns, *p* ≥ 0.05; *p < 0.05; **p < 0.01; ***p < 0.001

CD4⁺ T-cell subpopulations were isolated and subjected to dimensionality reduction, leading to the identification of nine distinct subsets (Fig. 5A). A t-SNE projection (Fig. 5B) and heatmaps of protein expression correlations (Fig. 5C) were used to delineate the phenotypic characteristics of these subsets. Inter-sample correlations based on CD4⁺ T-cell subset proportions were visualized in a hierarchical dendrogram (Fig. 5D). Compared with HCs, patients with MMD exhibited a significant reduction in the proportions of CD4 T01, T02, T06, and T07 subsets, and a notable increase in the proportions of CD4 T04, T05, T08, and T09 subsets (Fig. 5E).

**Fig. 6:**
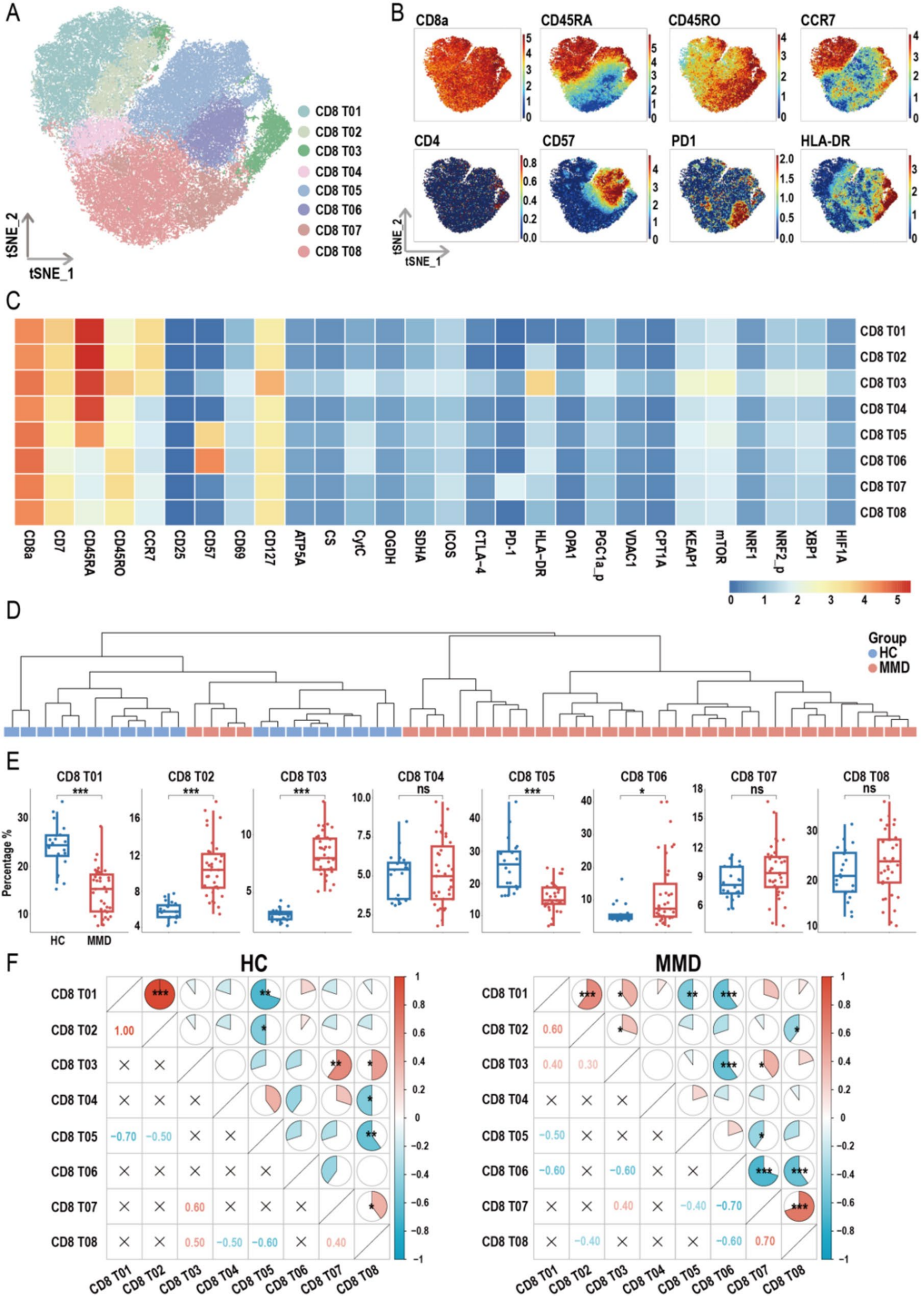
Overview of CD8^+^ T cell subsets. (**A**) t-SNE plot showing the distribution of CD8^+^ T cell subsets. (**B**) t-SNE heatmap displaying the expression levels of specific marker molecules in CD8^+^ T cell subsets. (**C**) Heatmap illustrating the correlation between CD8^+^ T cell subsets and their protein expression profiles measured by mass cytometry. (**D**) Cluster dendrogram based on the proportions of CD8^+^ T cell subsets. (**E**) Box plots comparing the proportions of CD8^+^ T cell subsets between healthy controls (HCs) and moyamoya disease (MMD) patients (*p*-values were calculated using the Wilcoxon rank-sum test). (**F**) Heatmap showing the intra-group correlations of CD8^+^ T cell subset proportions within the HC and MMD groups (*p*-values were calculated using Pearson correlation analysis). Significance: ns, *p* ≥ 0.05; *p < 0.05; **p < 0.01; ***p < 0.001

In addition to CD4⁺ T cells, CD8⁺ T-cell subpopulations were also isolated and subjected to dimensionality reduction, resulting in the identification of eight distinct subsets (Fig. 6A). Each subset was characterized through t-SNE projection (Fig. 6B) and heatmaps of protein expression correlations (Fig. 6C). Inter-sample correlations based on CD8⁺ T-cell subset distributions were visualized using a hierarchical dendrogram (Fig. 6D). Compared with HCs, patients with MMD exhibited a significant reduction in the proportions of CD8 T01 and CD8 T05 subsets, and a notable increase in CD8 T02, T03, and T06 subsets (Fig. 6E). Correlation analysis revealed distinct interrelationships among CD4⁺ and CD8⁺ T-cell subsets between HC and MMD groups (Figs. 5F and 6F), indicating disease-associated shifts in T-cell subset coordination.

### Annotation and expression profiles of CD4⁺ and CD8⁺ T-cell subsets

Characterization of CD4⁺ and CD8⁺ T-cell subsets revealed distinct immunophenotypic features (Table 1). The CD4 T01 subset, representative of naïve T (Tn) cells, exhibited strong proliferative capacity and survival potential. The CD4 T02 subset, classified as transitional Tn cells, showed potential for further differentiation. The CD4 T03 subset was characterized as senescent or terminally differentiated cells, displaying high metabolic activity but limited proliferation ability, suggesting its involvement in sustained immune responses. The CD4 T04 subset consisted of highly activated Tn cells, while CD4 T05 and T08 subsets were annotated as central memory T (Tcm) cells with long-term survival potential. The CD4 T06 subset comprised effector memory T (Tem) cells, marked by high effector functionality and reduced proliferative potential. The CD4 T07, defined by elevated PD-1 expression, represented exhausted T cells. The CD4 T09 subset, characterized by CD25 expression, was identified as Treg cells.

**Table 1:** Comparison of PhenoGraph-derived clusters with manually gated T cell phenotypes

Cell lineage	PhenoGraph cluster	Cell phenotype
T cells	T cells	CD3^+^
CD4^+^ T cells	CD4^+^ T cells	CD4^+^
	CD4 T01	CD45RA^high^ CD45RO^−^CCR7^+^
	CD4 T02	CD45RA^low^ CD45RO^−^CCR7^+^
	CD4 T03	CD45RA^high^ CD45RO^+^CCR7^+^CD57^+^
	CD4 T04	CD45RA^high^ CD45RO^+^CCR7^+^HLA-DR^+^
	CD4 T05	CD45RA^low^ CD45RO^+^CCR7^+^
	CD4 T06	CD45RA^−^CD45RO^+^CCR7^−^CD57^+^
	CD4 T07	PD-1^+^
	CD4 T08	CD45RA^−^CD45RO^+^CCR7^+^
	CD4 T09	CD25^+^
CD8^+^ T cells	CD8^+^ T cells	CD8^+^
	CD8 T01	CD45RA^+^CD45RO^−^CCR7^+^HLA-DR^−^
	CD8 T02	CD45RA^+^CD45RO^−^CCR7^+^HLA-DR^low^
	CD8 T03	CD45RA^+^CD45RO^+^CCR7^+^HLA-DR^high^
	CD8 T04	CD45RA^+^CD45RO^−^CCR7^−^
	CD8 T05	CD45RA^+^CCR7^−^CD57^+^
	CD8 T06	CD45RA^−^CD45RO^+^CCR7^−^CD57^+^
	CD8 T07	PD-1^+^
	CD8 T08	CD45RA^−^CD45RO^+^CCR7^−^CD57^−^
DPT cells	DPT cells	CD4^+^CD8^+^
DNT cells	DNT cells	CD4^−^CD8^−^

Abbreviations: DNT, double-negative T; DPT, double-positive T

The CD8 T01 subset exhibited high CD45RA expression, low CD45RO expression, CCR7 positivity, and HLA-DR negativity, consistent with a CD8⁺ Tn phenotype. The CD8 T02 subset closely resembled CD8 T01 but showed lower HLA-DR expression. The CD8 T03 subset, representing terminally differentiated cells, co-expressed CD45RA, CD45RO, CD57, and HLA-DR. The CD8 T04 subset was identified as effector T (Te) cells, while the CD8 T05 subset represented activated Te cells, characterized by moderate expression of CD45RA and CD57. Both CD8 T06 and CD8 T08 subsets were annotated as Tem cells, with CD8 T06 being CD57-positive and CD8 T08 being CD57-negative. The CD8 T07 subset, defined by high PD-1 expression, was classified as exhausted CD8⁺ T cells. Protein expression profiles of all CD4⁺ and CD8⁺ T-cell subsets, assessed via mass cytometry, are presented in Figures S2–S6.

### Markers associated with disease progression

**Fig. 7:**
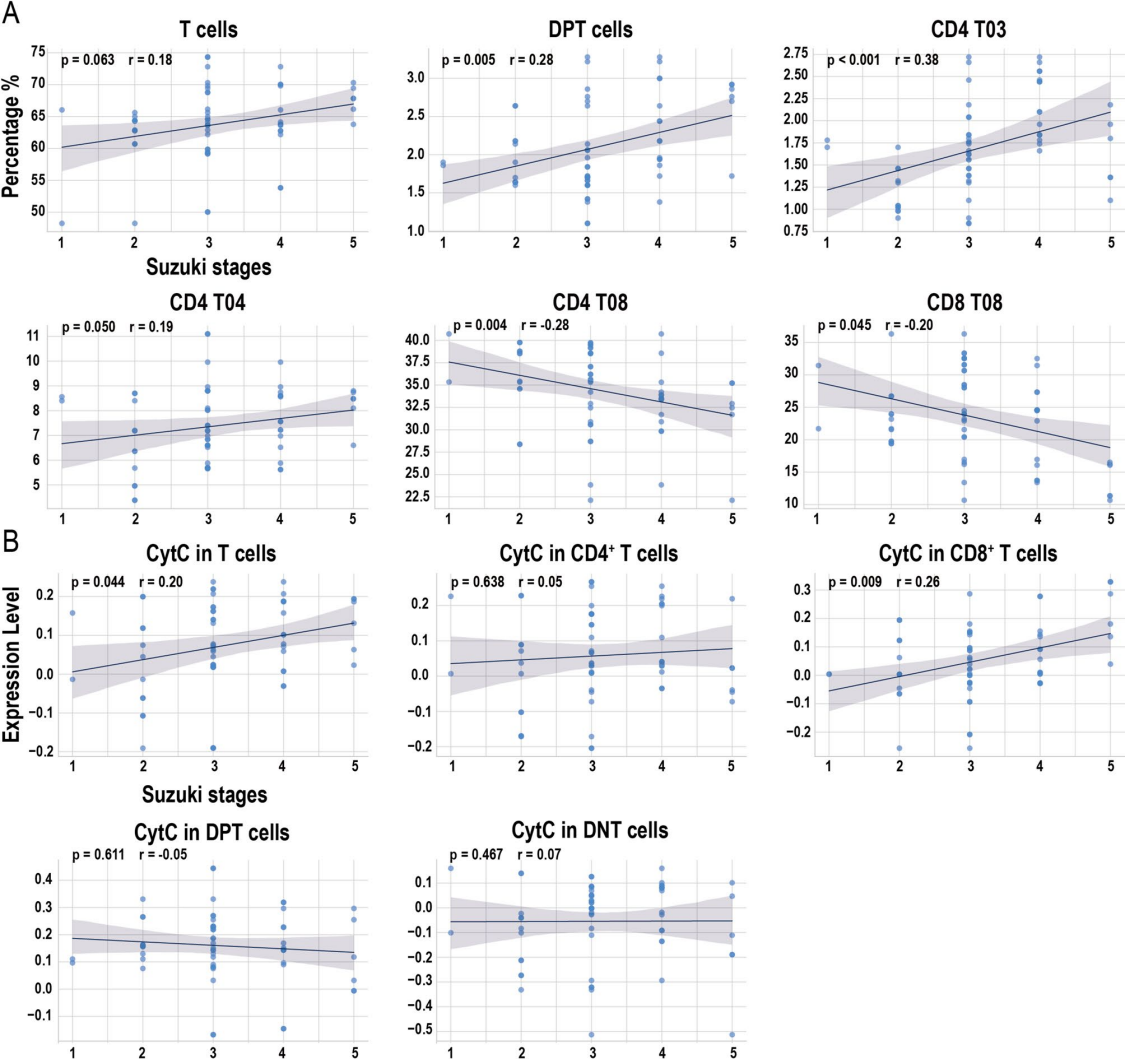
Mass cytometry analysis of cell subset proportions and cytochrome c (CytC) expression in relation to moyamoya disease (MMD) progression, as defined by Suzuki stages. (**A**) Proportions of total T cells and their subsets across Suzuki stages. (**B**) Expression levels of CytC in total T cells and their subsets. *P*-values were calculated using Spearman correlation analysis

The Suzuki stage is a widely accepted imaging-based metric used to assess the severity of MMD. As Suzuki stages increased, the proportions of total T cells, DPT cells, CD4 T03, and CD4 T04 subsets significantly increased, while the proportions of the CD4 T08 and CD8 T08 subsets significantly decrease (Fig. 7A). No significant linear correlations were observed between Suzuki stages and the proportions of other T-cell subsets (Figure S7). Furthermore, within both total T cells and CD8⁺ T-cell populations, CytC protein levels increased significantly with higher Suzuki stages, whereas no significant changes were observed in other subsets (Fig. 7B).

### Bulk transcriptomic analysis of PBMCs in MMD patients reveals key differential gene expression and pathway changes

**Fig. 8:**
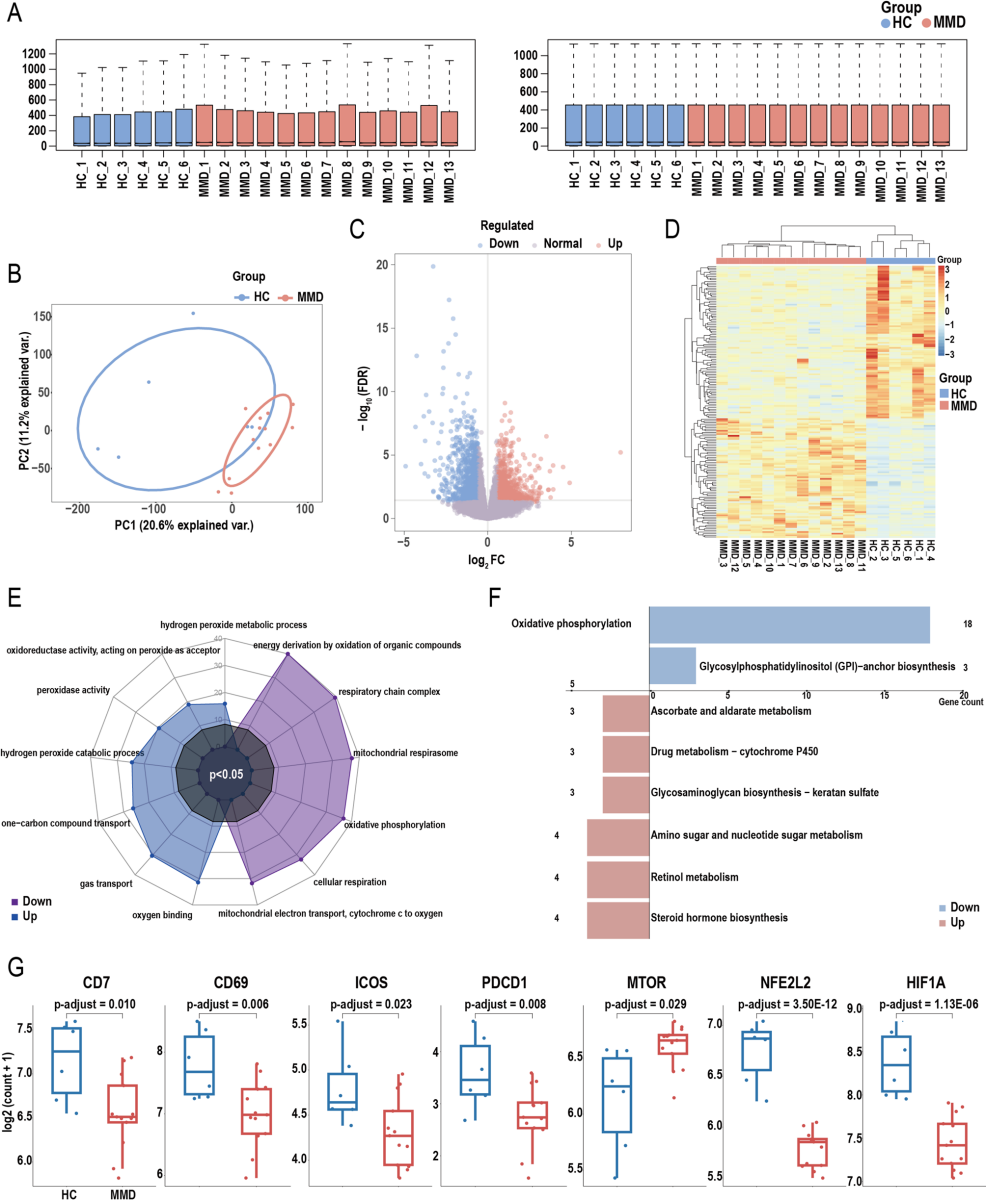
Bulk-seq analysis of peripheral blood mononuclear cells (PBMCs). (**A**) Workflow of data preprocessing and quality control. (**B**) Principal component analysis (PCA) showing dimensionality reduction and separation between healthy controls (HCs) and moyamoya disease (MMD) patients. (**C**) Volcano plot showing differentially expressed genes (DEGs) between HC and MMD groups. (**D**) Heatmap showing hierarchical clustering of samples based on the top 100 DEGs. (**E**) Gene Ontology (GO) enrichment analysis of DEGs between HC and MMD groups. (**F**) Kyoto Encyclopedia of Genes and Genomes (KEGG) enrichment analysis of DEGs between HC and MMD groups. (**G**) Comparison of RNA expression levels of genes associated with mass cytometry markers between HC and MMD groups, adjusted *p*-values were calculated using the Benjamini–Hochberg method implemented in the DESeq2 package in R

Given the high proportion of T cells among peripheral PBMCs, bulk RNA sequencing of PBMCs was performed to indirectly validate T cell–related immune signatures. The study cohort included 13 patients with MMD and 6 age- and sex-matched HCs, with baseline characteristics summarized in Table S3. Figure 8A shows the data distribution before and after normalization, while Fig. 8B illustrates intergroup separation following PCA-based dimensionality reduction. Differential gene expression analysis identified 941 upregulated and 1,047 downregulated genes in MMD patients compared to HCs, as visualized in the volcano plot (Fig. 8C). A heatmap of hierarchical clustering based on the top 100 upregulated and downregulated genes was generated to depict expression patterns and inter-sample relationships (Fig. 8D).

GO enrichment analysis revealed that upregulated genes in the MMD group were significantly associated with pathways related to oxygen binding, gas transport, and hydrogen peroxide metabolic process, among others. Conversely, downregulated genes were significantly enriched in pathways involving energy derivation by oxidation of organic compounds, respiratory chain complex, mitochondrial respirasome, oxidative phosphorylation, cellular respiration, and mitochondrial electron transport from cytochrome c to oxygen (Fig. 8E). KEGG pathway analysis further indicated that upregulated metabolic pathways in MMD group included retinol metabolism, steroid hormone biosynthesis, amino sugar and nucleotide sugar metabolism, ascorbate and aldarate metabolism, among others. In contrast, downregulated pathways were primarily enriched in oxidative phosphorylation and glycosaminoglycan biosynthesis–keratan sulfate (Fig. 8F).

At the transcriptomic level, the MMD group exhibited significantly decreased mRNA expression of CD7, CD69, ICOS, PDCD1 (encoding PD-1), NFE2L2 (encoding NRF2), and HIF1A, compared with the HC group. In contrast, MTOR expression was significantly upregulated in the MMD group (Fig. 8G). No other genes corresponding to proteins measured by mass cytometry showed statistically significant differences in mRNA expression between the two groups (Figure S8).

### Single-cell transcriptomic analysis of T-cell subpopulations in MMD patients reveals immune and metabolic pathway alterations

**Fig. 9:**
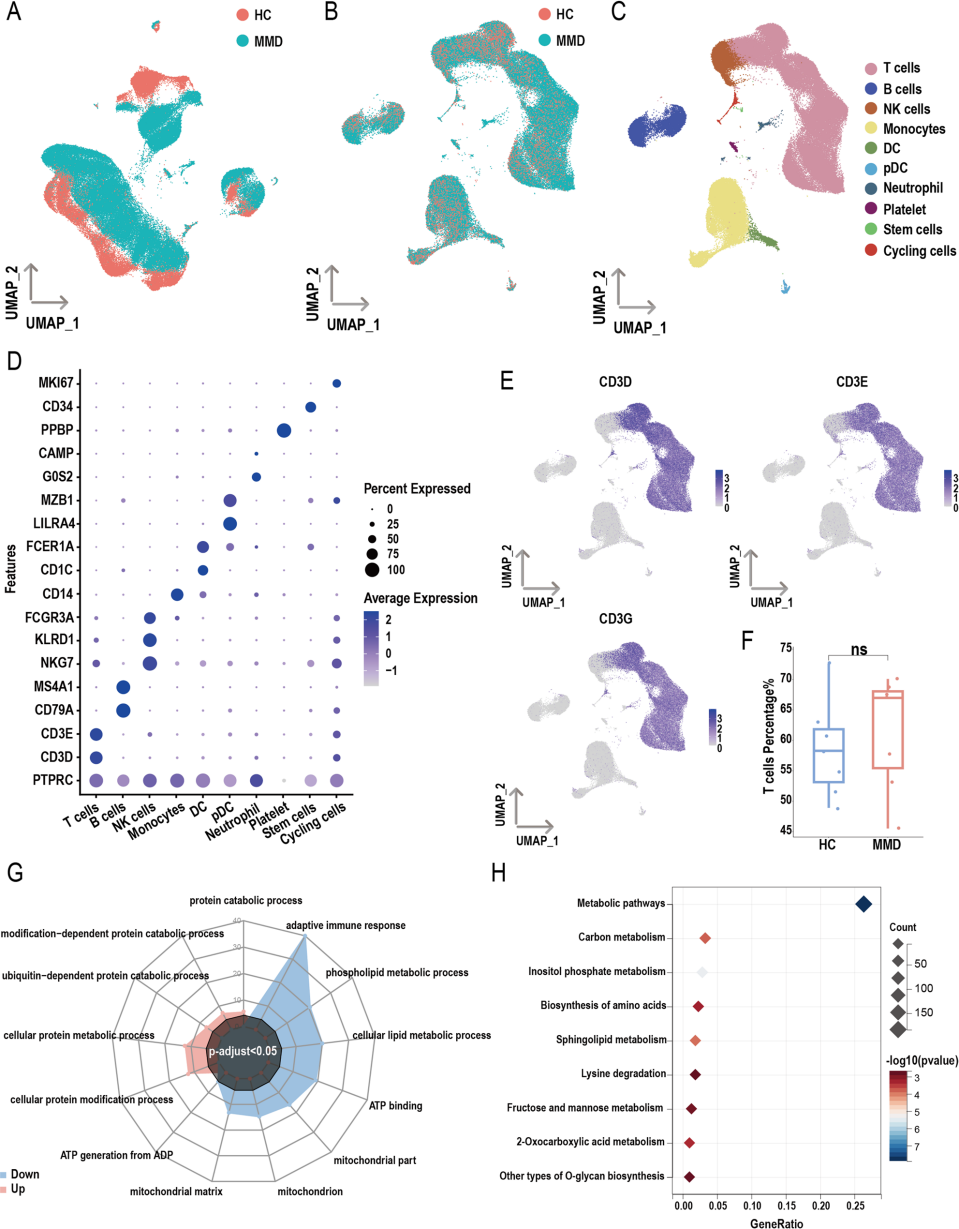
Single-cell RNA-seq analysis of peripheral blood mononuclear cells (PBMCs). (**A**) UMAP plot showing the distribution of cells between healthy controls (HCs) and moyamoya disease (MMD) patients before batch correction. (**B**) UMAP plot showing the distribution of cells after batch correction. (**C**) UMAP plot of dimensionality reduction for PBMCs. (**D**) Heatmap displaying PBMC subsets and cluster-specific marker expression. (**E**) UMAP plots showing the expression of CD3D, CD3E, and CD3G. (**F**) Box plot comparing the proportion of T cells between HC and MMD groups (*p*-values were calculated using the Wilcoxon rank-sum test). (**G**) Gene Ontology (GO) enrichment analysis of differentially expressed genes (DEGs) in T cells between HC and MMD groups. (**H**) Kyoto Encyclopedia of Genes and Genomes (KEGG) enrichment analysis of downregulated DEGs in T cells between HC and MMD groups

To further validate our findings, we analyzed publicly available single-cell RNA sequencing datasets from 7 HCs and 7 patients with MMD, with baseline characteristics summarized in Table S4. Figure 9A displays the UMAP projection of cells from both groups prior to batch correction, while Fig. 9B shows the UMAP plot following correction. Annotated subpopulations after dimensionality reduction are presented in Fig. 9C, and representative marker gene expression for each cluster is visualized in Fig. 9D. T-cell subpopulations were identified based on UMAP heatmaps of CD3D, CD3E, and CD3G expression (Fig. 9E), which revealed no significant differences in T-cell subset proportions between HC and MMD groups (Fig. 9F).

GO enrichment analysis revealed that, compared with the HC group, upregulated genes in the MMD group were significantly associated with pathways involved in cellular protein modification process, cellular protein metabolic process, ubiquitin-dependent protein catabolic process, modification-dependent protein catabolic process, and protein catabolic process. Conversely, downregulated genes were significantly enriched in pathways related to the adaptive immune response, phospholipid metabolic process, cellular lipid metabolic process, ATP binding, mitochondrial part, mitochondrion, mitochondrial matrix, and ATP generation from ADP (Fig. 9G). KEGG pathway analysis revealed that downregulated genes in the MMD group were significantly associated with metabolic pathways, carbon metabolism, inositol phosphate metabolism, biosynthesis of amino acids, sphingolipid metabolism, lysine degradation, and fructose and mannose metabolism, among others (Fig. 9H).

Gene Set Enrichment Analysis (GSEA) revealed significant downregulation of the T-cell receptor complex and PD-1 signaling pathway in the MMD group (Figures S9A-B). Following further dimensionality reduction of isolated T-cell populations, five distinct T-cell subsets were identified (Figure S9C) with their representative marker genes for each subset shown in Figure S9D. Compared to the HC group, the MMD group exhibited a significant increase in CD4⁺ T-cell subsets and a significant decrease in CD8⁺ T-cell subsets, with no significant differences observed in the proportions of the remaining subsets (Figure S9E).

## Discussion

In this study, we comprehensively characterized peripheral T-cell populations and their associated metabolic pathways in PBMCs from patients with MMD using mass cytometry combined with transcriptomics. Our findings revealed significant alterations in T-cell activation states and subset distribution in MMD, accompanied by mitochondrial dysfunction and compromised oxidative phosphorylation. Additionally, we observed marked elevations in intracellular oxidative stress and endoplasmic reticulum stress, alongside a disease-specific reduction in the expression of immune checkpoint molecules PD-1 and ICOS. These results underscore the critical contribution of immunometabolic dysregulation to the pathogenesis of MMD, and offer novel mechanistic insights into the disease at the intersection of immune activation, cellular metabolism, and cerebrovascular pathology.

T cells play a central role in the pathogenesis of autoimmune diseases by initiating and sustaining inflammatory immune responses. Through the secretion of pro-inflammatory cytokines, they influence both immune and non-immune cell populations, ultimately contributing to tissue damage [33–35]. In MMD, previous studies have reported elevated levels of Th17-associated cytokines—including interleukin (IL)-17, TNF-α, IL-6, and IL-23—as well as Treg-associated cytokines such as TGF-β and IL-10 [15]. Notably, peripheral blood analyses have revealed significantly increased levels of granulocyte-macrophage colony-stimulating factor (GM-CSF), a cytokine primarily secreted by Th1 and Th17 cells. Elevated GM-CSF promotes monocyte activation and enhances the secretion of angiogenic, inflammatory, and chemotactic cytokines, thereby contributing to the formation of abnormal vascular networks in MMD [36]. To further investigate T-cell activation and differentiation states in MMD, we assessed the expression of several established markers: CD45RO (memory T cells) [37], CCR7 (T-cell chemotaxis) [38], CD25 (activated T cells/Tregs) [39], CD69 (early activation) [40], CD127 (mature T cells, particularly Tcm cells) [41], and HLA-DR (antigen presentation/activation) [42]. Although the overall T-cell proportion did not differ significantly between MMD patients and HCs, patients with MMD exhibited markedly increased expression of CD45RO, CCR7, CD25, CD69, CD127, and HLA-DR, alongside reduced CD45RA expression. These results suggest that T cells in MMD adopt a highly activated, memory-like phenotype, potentially driven by persistent antigenic stimulation. Sustained production of pro-inflammatory cytokines by these activated T cells may play a pivotal role in disease pathogenesis.

CD4⁺ T cells play a central role in adaptive immunity by secreting cytokines, promoting B-cell antibody production, and modulating the cytotoxic functions of CD8⁺ T cells [43]. In our study, mass cytometry combined with single-cell transcriptomic validation revealed a significant increase in the proportion of CD4⁺ T cells and a concurrent decrease in CD8⁺ T cells in patients with MMD. The expansion of CD4⁺ T cells suggests an enhanced helper T-cell–driven inflammatory response, whereas the reduction in CD8⁺ T cells may indicate impaired cytotoxic immune surveillance. Importantly, this increase in CD4^+^ T cells does not represent a global enhancement of adaptive immunity. On the contrary, single-cell analysis demonstrated overall downregulation of general T cell–mediated adaptive immune pathways in MMD, highlighting a dysregulated immune landscape rather than a functionally strengthened response.

Further analysis of CD4⁺ and CD8⁺ T-cell subsets revealed complex phenotypic alterations in MMD. Among CD4⁺ T cells, there was a notable reduction in resting and transitional Tn cells, as well as Tem cells, accompanied by an increase in activated Tn and Tcm cells. A similar pattern was observed among CD8⁺ T-cell subsets, with decreases in Tn and Te cells, and increases in activated Tn cells and CD57⁻ Tem cells. Additionally, Tregs (CD4 T09) were increased. Although the expansion of Tregs may reflect a compensatory mechanism aimed at counteracting excessive T-cell activation and restoring immune homeostasis, our previous research suggests that these Tregs are fragile and functionally impaired [17]. Beyond their well-established immunoregulatory role, Tregs are now recognized for their influence on vascular physiology, regulating endothelial cell proliferation and apoptosis through multiple signaling pathways critical for stable angiogenesis [44]. Overall, both CD4⁺ and CD8⁺ T cells in MMD exhibit a dynamic shift from “resting/transitional” to “activated/memory” and ultimately “terminally differentiated” states. This redistribution of T-cell subsets reflects an underlying immune imbalance in MMD patients, indicative of chronic inflammatory or excessive immune activation.

The Suzuki stage is a widely used imaging-based scoring system in clinical practice for assessing the severity and progression of MMD, reflecting cerebral perfusion in the anterior circulation [45]. With increasing Suzuki stage, anterior circulation perfusion declines, leading to cerebral ischemia. This ischemic environment triggers a pro-inflammatory cascade that leads to neuronal cell death. Necrotic neurons release damage-associated molecular patterns (DAMPs), which activate macrophages. In turn, activated macrophages secrete pro-inflammatory cytokines and recruit T cells, thereby amplifying the local immune response [46, 47]. In our study, higher Suzuki stages were associated with increased proportions of terminally differentiated CD4⁺ T cells and activated CD4⁺ Tn cells, accompanied by a decline in CD4⁺ Tcm cells and CD8⁺ CD57⁺ Tem cells. These alterations suggest a pattern of chronic or repeated antigenic stimulation that drives T cells populations toward progressive activation and terminal differentiation. Under moderate stimulation, Tcm cells typically differentiate into Tem cells; however, sustained or excessive stimulation, as seen in advanced disease stages, may lead to terminal differentiation or functional exhaustion, diminishing their functional capacity [48]. Notably, CD57⁺ expression is a marker of high cytotoxicity but limited proliferative potential. The observed decline in CD8⁺ CD57⁺ Tem cells in patients with advanced-stage MMD may reflect their rapid depletion or clearance within a highly activated immune microenvironment [49]. As disease severity increases, the Tcm cell pool may become insufficiently maintained, resulting in a more robust but less adaptable immune response. This shift could ultimately contribute to greater vascular injury and impaired repair mechanisms in MMD.

In the thymus, developing T cells transition from DNT to DPT cells before undergoing lineage commitment into CD4⁺ or CD8⁺ single-positive subsets, which subsequently enter peripheral circulation [50]. Compared with healthy individuals, patients with MMD exhibit a higher proportion of circulating DPT cells, and this proportion increases progressively with higher Suzuki stages. This observation suggests the possibility of impaired thymic selection or dysregulated peripheral homeostasis during MMD progression, however, further mechanistic studies are needed to confirm this hypothesis. Notably, the proportion of DPT cells may represent a novel cellular biomarker for both the onset and progression of MMD. Additionally, we observed a positive correlation between CytC levels and Suzuki stages in both total T cells and CD8⁺ T-cell subsets. CytC is closely linked to oxidative stress; exposure to peroxides, radiation, or other oxidizing agents can induce mitochondrial damage, leading to the release of CytC into the cytoplasm and subsequent activation of apoptosis pathways [51, 52]. Although we did not observe a reduction in total T-cell or CD8⁺ T-cell proportions consistent with CytC-induced apoptosis, our findings suggest that intracellular accumulation of CytC in these populations may serve as an independent indicator of disease progression.

In both acute and chronic infections, T cells undergo functional exhaustion following antigenic stimulation, often characterized by the upregulation of immune checkpoint molecules [53, 54]. High-affinity T-cell receptor signaling is a critical driver in the generation of exhausted T-cell precursors, while the PD-1 signaling axis serves as a key inhibitory mechanism to limit excessive immune activation [55]. However, our data do not support the presence of T-cell exhaustion in patients with MMD. At the transcriptional level, PDCD1 (encoding PD-1) was downregulated in MMD, and single-cell transcriptomic analysis revealed reduced expression of both the T cell receptor complex and the PD-1 signaling pathway. Consistently, protein-level analysis showed decreased expression of CD3 and PD-1. Within the CD4⁺ T-cell subsets, the proportion of PD-1⁺ cells (CD4 T07 subset) was lower in MMD patients compared with HCs. Taken together, these findings suggest that rather than entering a state of exhaustion, T cells in MMD may be transcriptionally regulated to favor sustained activation. A similar phenomenon has been reported in a hypoxic mouse model of pulmonary hypertension, where PD-1 downregulation in Th17 cells and PD-L1 downregulation in pulmonary endothelial cells were observed [56]. This raises the possibility that PD-1/PD-L1 axis dysregulation in MMD may be associated with neural hypoxia or may reflect a metabolic imbalance arising from persistent T-cell activation under limited intracellular oxygen availability.

CTLA-4 is a negative regulatory molecule that is primarily expressed upon T-cell activation and functions to suppress excessive T-cell responses by competing with CD28 for binding to B7 family ligands [57, 58]. In patients with MMD, CTLA-4 expression was upregulated in CD4⁺ T cells but downregulated in CD8⁺ T cells, suggesting that CTLA-4 may serve to limit CD4^+^ T-cell activation and thereby mitigate pro-inflammatory cytokine release. Conversely, the reduced CTLA-4 expression on CD8⁺ T cells may contribute to their increased activation. Interestingly, ICOS—a co-stimulatory molecule typically associated with enhanced T-cell activation—was unexpectedly downregulated at the protein level in both total T cells and their subsets [59]. This observation appears inconsistent with the overall activated T-cell phenotype in MMD, indicating potential post-transcriptional regulation or altered receptor turnover. Further investigation is warranted to clarify the mechanisms underlying this discrepancy. In conclusion, the dysregulation of T-cell immune checkpoints in MMD reflects an imbalance between co-stimulatory and inhibitory signaling pathways. This imbalance likely lowers the threshold for T-cell activation, predisposing patients to heightened immune activation and unchecked inflammatory responses.

To support the dynamic transition between immune cell subsets and sustain T-cell effector function, metabolic enzymes and energy utilization pathways must undergo adaptive changes—a process known as metabolic reprogramming [60, 61]. In MMD, evidence of such reprogramming was observed in peripheral T cells. Transcriptomic analysis of PBMCs revealed that upregulated pathways in the MMD group were significantly enriched in oxygen-binding and hydrogen peroxide metabolism-related processes, while mitochondrial- and oxidative phosphorylation–related pathways were markedly downregulated. Single-cell transcriptomic profiling of T-cell subsets further demonstrated a metabolic shift characterized by increased reliance on protein catabolism and decreased lipid metabolism. Despite the presence of activated T-cell phenotypes, mitochondrial dysfunction emerged as another key finding. Specifically, expression of OPA1 and VDAC1—proteins critical for mitochondrial dynamics—was significantly reduced in T cells from MMD patients. Key TCA cycle enzymes, including ATP5A and CS, were also downregulated, while CytC was upregulated. Additionally, OGDH expression was decreased in both CD4⁺ and CD8⁺ T-cell subsets. Collectively, these changes suggest that mitochondrial respiratory chain activity and overall bioenergetic metabolism are impaired in MMD T cells, which may compromise cellular function and contribute to immune dysregulation in disease pathogenesis.

Mitochondrial dysfunction in T cells not only compromises energy production but also enhances the generation of reactive oxygen species (ROS), which can induce apoptosis or functional impairment, ultimately threatening immune cell survival [62]. Transcriptomic analyses of peripheral PBMCs in MMD revealed upregulation of hydrogen peroxide metabolism—a key ROS-related process Hydrogen peroxide, a major form of ROS, can accumulate intracellularly and generate highly reactive hydroxyl radicals, leading to oxidative damage and disruption of cellular functions [63]. Excessive ROS contributes to oxidative stress, a pathological driver of inflammation, aging, and immune dysregulation [64]. In response to impaired oxidative phosphorylation, T cells may shift toward glycolysis, a metabolic signature frequently associated with pro-inflammatory states [65, 66]. This reprogramming could exacerbate vascular inflammation and accelerate MMD progression. Moreover, the upregulation of stress-related pathways—including KEAP1 and XBP1—suggests that T cells in MMD experience both oxidative and endoplasmic reticulum stress [67, 68]. These stress responses can reshape gene expression and cellular metabolism, intensifying inflammation and vascular pathology. Notably, aberrant regulation of the KEAP1–NRF2 axis may compromise antioxidant defense mechanisms [69], thereby amplifying oxidative damage and inflammatory responses.

The dysregulation of T-cell immune metabolism in MMD may serve as a potential driver of disease progression, offering new biomarkers for early diagnosis. Clinically, a deeper understanding of the immune metabolic characteristics of MMD provides potential directions for novel therapeutic interventions. For instance, enhancing mitochondrial function or regulating oxidative phosphorylation could become new treatment strategies aimed at restoring immune balance and alleviating vascular inflammation. Furthermore, modulating specific T-cell subsets, such as enhancing the function of Treg cells or inhibiting the overactivation of effector T cells, may also have a positive impact on MMD treatment. Given the downregulation of the PD-1 signaling pathway, the potential of immune checkpoint modulators in MMD management warrants further attention.

### Limitations and future directions

This study has several limitations. First, the relatively small sample size—particularly in the RNA-seq cohort—may limit statistical power and reduce sensitivity for detecting DEGs and enriched pathways. As such, the transcriptomic findings should be considered exploratory and require validation in larger, independent cohorts. Second, although immune and mitochondrial dysfunction are central themes in the pathogenesis of MMD, this study did not assess *RNF213* genotyping, including the common East Asian variant p.R4810K. The lack of genetic stratification limits the ability to interpret gene–immune–metabolic interactions and may reduce the generalizability of our results across different genetic backgrounds. Finally, although the discussion proposes several mechanistic hypotheses based on the current findings, these interpretations remain speculative and require further validation through multicenter studies with larger sample sizes and integrated genetic and functional profiling.

## Conclusions

This study systematically highlights significant alterations in peripheral T-cell subsets and their metabolic profiles in patients with MMD, emphasizing the pivotal role of immune-metabolic dysregulation in disease pathogenesis. The observed shifts in T-cell differentiation, activation, and mitochondrial function provide mechanistic insights into the immune landscape of MMD and point to potential therapeutic targets for future intervention.

## Electronic supplementary material

Below is the link to the electronic supplementary material.

Supplementary Material 1

## Data Availability

The datasets generated and/or analyzed during the current study are available from the corresponding author upon reasonable request.

## Abbreviations

ATP5A: ATP synthase subunit alpha, mitochondrial
bp: Base pair
CCR7: C-C Chemokine Receptor Type 7
CPT1A: Carnitine Palmitoyltransferase1A
CPM: Counts Per Million
CS: Citrate Synthase
CTLA-4: Cytotoxic T-Lymphocyte Protein 4
CytC: Cytochrome C
DAMPs: Damage-Associated Molecular Patterns
DEGs: Differentially Expressed Genes
DMSO: Dimethyl Sulfoxide
DNT: Double-Negative T Cells
DPT: Double-Positive T Cells
DSA: Digital Subtraction Angiography
ETC: Electron Transport Chain
FBS: Fetal Bovine Serum
FDR: False Discovery Rate
GM-CSF: Granulocyte-Macrophage Colony-Stimulating Factor
GO: Gene Ontology
GSEA: Gene Set Enrichment Analysis
HC: Healthy Control
HIF1A: Hypoxia-Inducible Factor 1-Alpha
HLA-DR: Human Leukocyte Antigen–DR Isotype
ICOS: Inducible T-Cell Costimulator
IDH: Isocitrate Dehydrogenase
IL: Interleukin
KEAP1: Kelch-like ECH-Associated Protein 1
KEGG: Kyoto Encyclopedia of Genes and Genomes
MMD: Moyamoya Disease
mTOR: Mammalian Target of Rapamycin
NRF1: Nuclear Respiratory Factor 1
NRF2_p: Phosphorylated Nuclear Factor Erythroid 2–Related Factor 2
OGDH: Oxoglutarate Dehydrogenase
OPA1: Optic Atrophy 1
PBMCs: Peripheral Blood Mononuclear Cells
PBS: Phosphate-Buffered Saline
PCA: Principal Component Analysis
PD-1: Programmed Cell Death Protein 1
PGC1a_p: Phosphorylated Peroxisome Proliferator-Activated Receptor Gamma Coactivator 1-Alpha
RBC: Red Blood Cell
RNA-seq: RNA sequencing
ROS: Reactive oxygen species
SD: Standard Deviation
SDHA: Succinate Dehydrogenase Complex Flavoprotein Subunit A
TCA: Tricarboxylic Acid Cycle
Tcm: Central Memory T Cells
Te: Effector T Cells
Tem: Effector Memory T Cells
Th17: T helper 17 cells
Tn: Naïve T Cells
TOMM7: Translocase of the Outer Mitochondrial Membrane 7
Treg: Regulatory T Cells
t-SNE: t-distributed Stochastic Neighbor Embedding
VDAC1: Voltage-Dependent Anion-Selective Channel Protein 1
XBP1: X-Box Binding Protein 1
