# Supplementary material for: Circulating T cell atlas in Moyamoya disease: insights into immunopathogenesis of cerebrovascular disorders
Source: J Neuroinflammation. 2025 Jun 5;22:151. doi: 10.1186/s12974-025-03479-3 (PMC12142972; doi:10.1186/s12974-025-03479-3)
Supplement: Supplementary file 1 — Supplementary Material 1 [file 12974_2025_3479_MOESM1_ESM.docx]

Supplementary Material

**Supplementary Table 1. Detailed panel of antibodies employed in mass cytometry.**

| Antigen | Symbol and Mass | Clone | Source |
| --- | --- | --- | --- |
| CD45 | 89Y | HI30 | Fluidigm |
| CD4 | 116Cd | RPA-T4 | BioLegend |
| CS | 141Pr | EPR8067 | Abcam |
| CD14 | 142Ce | 134620R | R&D |
| CD11c | 143Nd | ICRF 3.9 | R&D |
| CPT1A | 144Nd | 8F6AE9 | Abcam |
| ATP5A | 145Nd | 15H4C4 | Abcam |
| CD8a | 146Nd | RPA-T8 | Fluidigm |
| CD7 | 147Sm | CD7-6B7 | Fluidigm |
| CD45RO | 148Sm | CuHL1 | BioLegend |
| CD25 | 149Sm | 2A3 | Fluidigm |
| SDHA | 150Sm | 2E3GC12FB2AE2 | Abcam |
| CD16 | 151Eu | 3G8 | BioLegend |
| OGDH | 152Gd | EPR27181-78 | Abcam |
| OPA1 | 153Eu | 1E81D9 | Abcam |
| VDAC1 | 154Gd | 20B12AF2 | Abcam |
| PD-1 | 155Gd | EH12.2H7 | Fluidigm |
| PGC1a_p | 156Gd | S571 | R&D |
| IDH | 158Gd | EPR21002 | Abcam |
| CCR7 | 159Tb | G043H7 | Fluidigm |
| NRF1 | 160Dy | EPR5554(N) | Abcam |
| CTLA-4 | 161Dy | 14D3 | Fluidigm |
| CD69 | 162Dy | FN50 | Fluidigm |
| NRF2_p | 163Dy | EP1809Y | Fluidigm |
| KEAP1 | 164Dy | EPR22664-26 | Abcam |
| HIF1A | 166Er | BL-124-3F7 | Abcam |
| mTOR | 167Er | Y391 | Abcam |
| ICOS | 168Er | C398.4A | Fluidigm |
| CD45RA | 169Tm | HI100 | Fluidigm |
| CD3 | 170Yb | UCHT1 | Fluidigm |
| XBP1 | 171Yb | 143F | Abcam |
| CD57 | 172Yb | HCD57 | Fluidigm |
| CytC | 173Yb | 6H2.B4 | BioLegend |
| HLA-DR | 174Yb | LN3 | BioLegend |
| CD19 | 175Lu | HIB19 | Fluidigm |
| CD127 | 176Lu | A019D5 | Fluidigm |

| Characteristics | MMD patients (N = 35) | HCs  (N = 20) | *p*-value |
| --- | --- | --- | --- |
| Demographic |  |  |  |
| Age (years), mean ± SD | 38.4 ± 7.1 | 35.4 ± 7.7 | 0.151 |
| Female/male ratio | 17/18 | 11/9 | 0.646 |
| Vascular risk factors, n (%) |  |  |  |
| Hypertension | 10 (28.6) | 0 (0.0) | 0.023* |
| Hyperlipidemia | 5 (14.3) | 0 (0.0) | 0.147 |
| Diabetes mellitus | 1 (2.9) | 0 (0.0) | 1.000 |
| Current smoking | 8 (22.9) | 0 (0.0) | 0.055 |
| Current alcohol consumption | 5 (14.3) | 0 (0.0) | 0.147 |
| Clinical manifestations, n (%) |  |  |  |
| Ischemic type | 20 (57.1) | - | - |
| Hemorrhagic type | 15 (42.9) | - | - |
| Suzuki stage, n (%) |  |  | - |
| Stage 1-2 | 7 (20.0) | - | - |
| Stage 3-4 | 22 (62.9) | - | - |
| Stage 5-6 | 6 (17.1) | - | - |

**Supplementary Table 2. Summary of clinical features of MMD patients and healthy controls analyzed by mass cytometry.**

Abbreviations: HCs, healthy controls; MMD, moyamoya disease; SD, standard deviation.

Statistical significance: **p* < 0.05.

**Supplementary Table 3. Summary of clinical characteristics for MMD patients and healthy controls in the bulk RNA-seq cohort.**

| Characteristics | MMD patients (N = 13) | HCs  (N = 6) | *p*-value |
| --- | --- | --- | --- |
| Demographic |  |  |  |
| Age (years), mean ± SD | 38.5 ± 7.5 | 37.3 ± 6.3 | 0.738 |
| Female/male ratio | 8/5 | 3/3 | 1.000 |
| Vascular risk factors, n (%) |  |  |  |
| Hypertension | 2 (15.4) | 0 (0.0) | 1.000 |
| Hyperlipidemia | 0 (0.0) | 0 (0.0) | - |
| Diabetes mellitus | 1 (7.7) | 0 (0.0) | 1.000 |
| Current smoking | 2 (15.4) | 0 (0.0) | 1.000 |
| Current alcohol consumption | 1 (7.7) | 0 (0.0) | 1.000 |
| Clinical manifestations, n (%) |  |  |  |
| Ischemic type | 6 (46.2) | - | - |
| Hemorrhagic type | 7 (53.8) | - | - |
| Suzuki stage, n (%) |  |  | - |
| Stage 1-2 | 1 (7.7) | - | - |
| Stage 3-4 | 10 (76.9) | - | - |
| Stage 5-6 | 2 (15.4) | - | - |

Abbreviations: HCs, healthy controls; MMD, moyamoya disease; SD, standard deviation.

**Supplementary Table 4. Clinical characteristics of patients with MMD and healthy controls in the scRNA-seq cohort.**

| Characteristics | MMD patients (N = 7) | HCs  (N = 7) |
| --- | --- | --- |
| Demographic |  |  |
| Age (years), mean ± SD | 41.9 ± 5.5 | 40.3 ± 5.8 |
| Female/male ratio | 3/4 | 3/4 |
| Vascular risk factors, n (%) |  |  |
| Hypertension | 2 (28.6) | - |
| Hyperlipidemia | 2 (28.6) | - |
| Diabetes mellitus | 0 (0.0) | - |
| Current smoking | 1 (14.3) | - |
| Current alcohol consumption | 1 (14.3) | - |
| Clinical manifestations, n (%) |  |  |
| Ischemic type | 4 (57.1) | - |
| Hemorrhagic type | 3 (42.9) | - |

Abbreviations: HCs, healthy controls; MMD, moyamoya disease; SD, standard deviation.

HCs data were obtained from the GSE165080 dataset, and MMD data were derived from HRA005203.


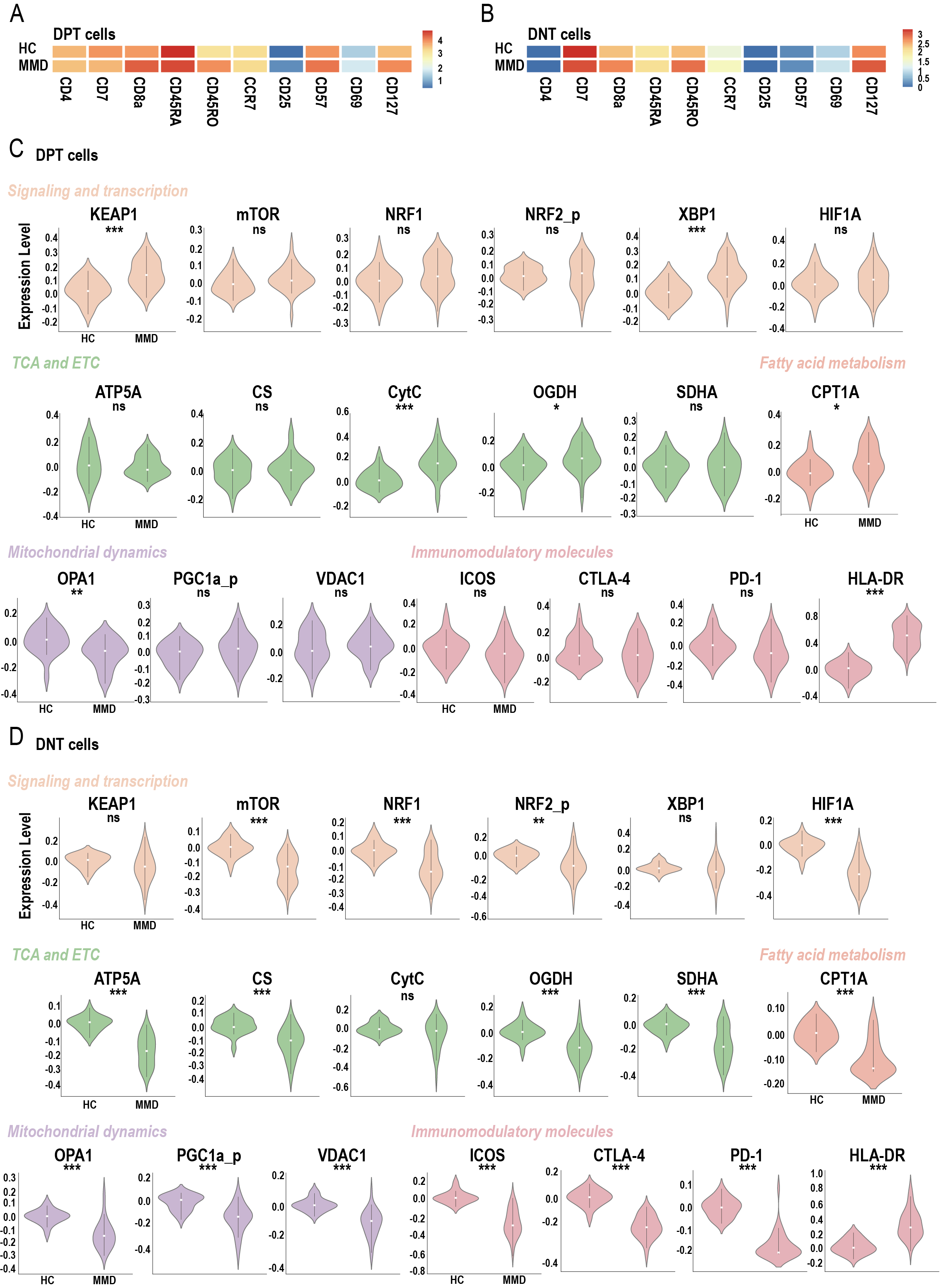


**Fig S1. Mass cytometry analysis of molecular expression differences in DPT and DNT cell subsets between healthy control (HC) and moyamoya disease (MMD) groups.** Heatmaps illustrating the expression of T-cell activation and differentiation markers in (A) DPT cells and (B) DNT cells between HC and MMD groups. Differential expression of metabolic and functional state markers analyzed by mass cytometry in (C) DPT cells and (D) DNT cells between HC and MMD groups. Statistical significance: ns, *p* ≥ 0.05; **p* < 0.05; ***p* < 0.01; ****p* < 0.001 (Wilcoxon rank-sum test).


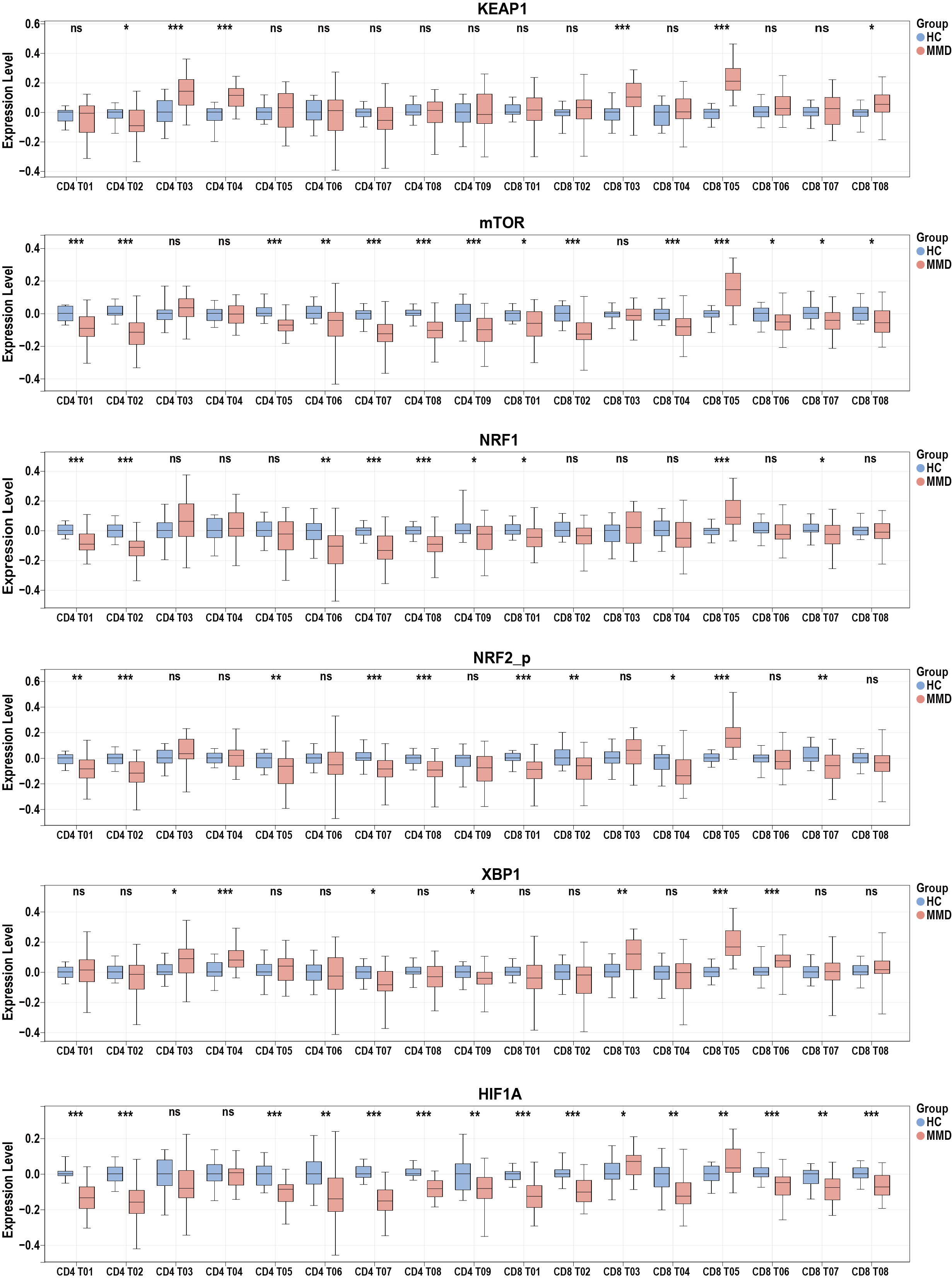


**Fig S2. Mass cytometry analysis of signaling– and transcription–related molecules in CD4^+^ and CD8^+^ T cell subsets between healthy control (HC) and moyamoya disease (MMD) groups.** Statistical significance: ns, *p* ≥ 0.05; **p* < 0.05; ***p* < 0.01; ****p* < 0.001 (Wilcoxon rank-sum test).


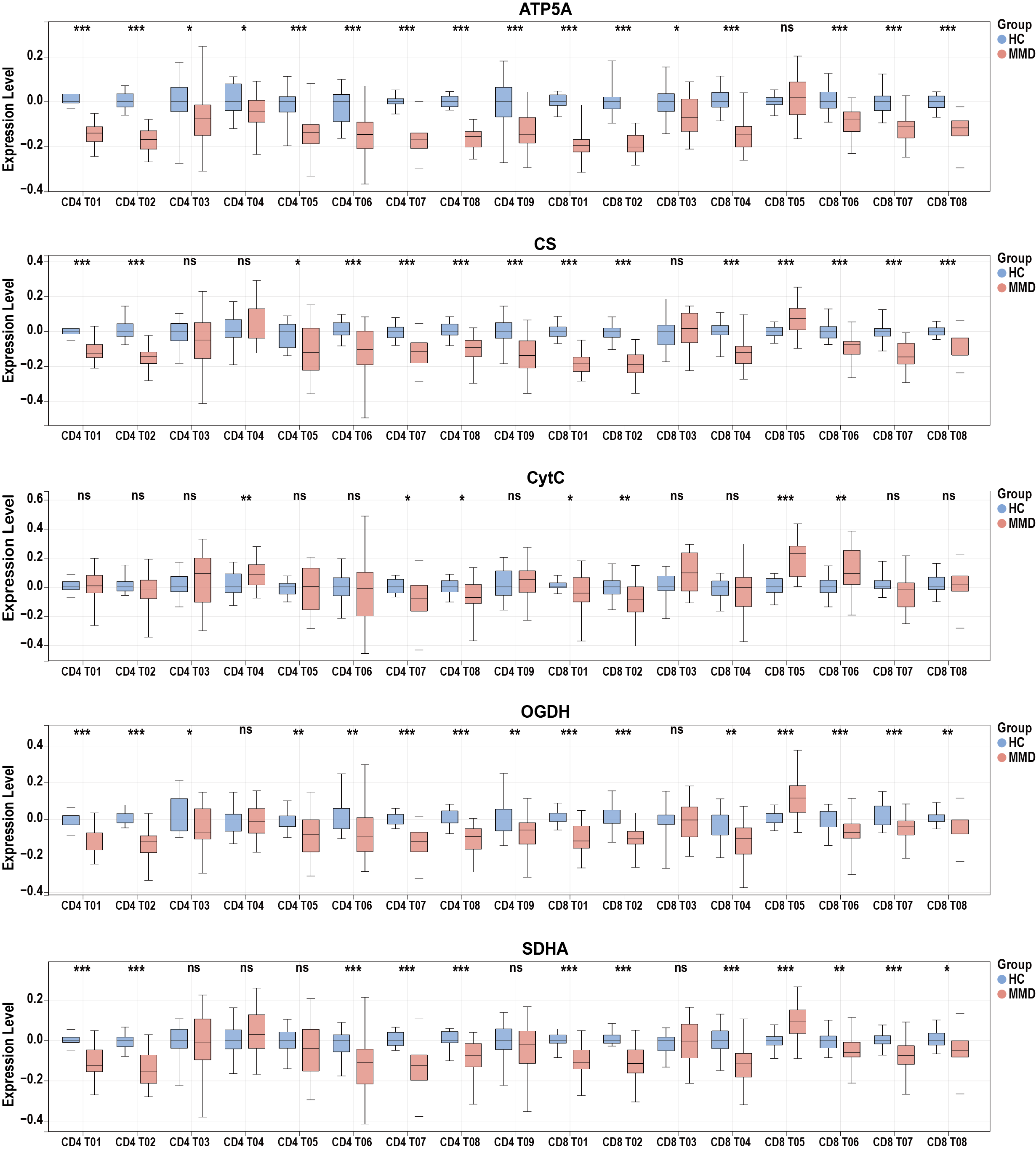


**Fig S3. Mass cytometry analysis of tricarboxylic acid (TCA) cycle– and electron transport chain (ETC)–related molecules in CD4^+^ and CD8^+^ T cell subsets between healthy control (HC) and moyamoya disease (MMD) groups.** Statistical significance: ns, *p* ≥ 0.05; **p* < 0.05; ***p* < 0.01; ****p* < 0.001 (Wilcoxon rank-sum test).


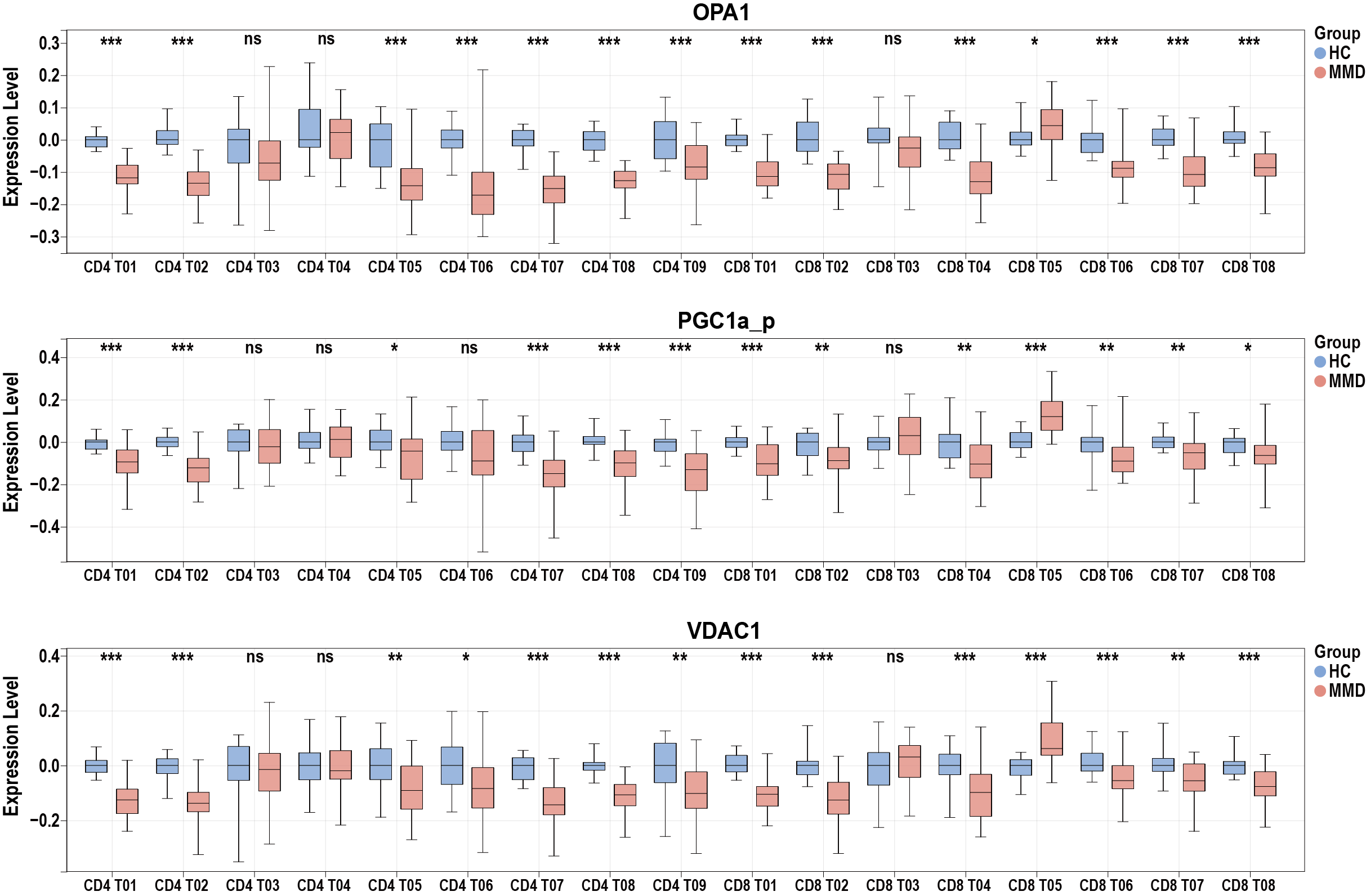


**Fig S4. Mass cytometry analysis of mitochondrial dynamics–related molecules in CD4^+^ and CD8^+^ T cell subsets between healthy control (HC) and moyamoya disease (MMD) groups.** Statistical significance: ns, *p* ≥ 0.05; **p* < 0.05; ***p* < 0.01; ****p* < 0.001 (Wilcoxon rank-sum test).


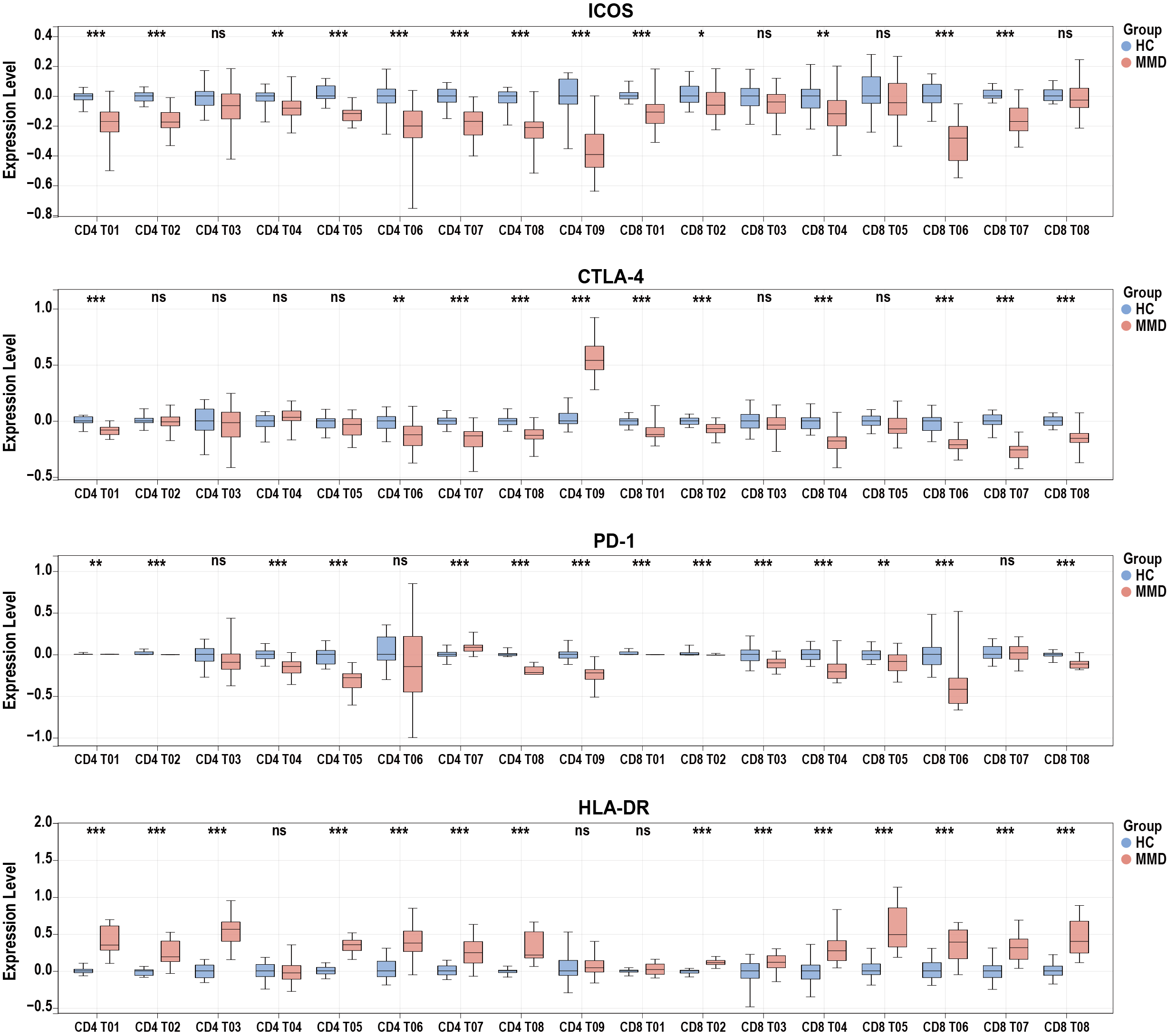


**Fig S5. Mass cytometry analysis of immunomodulatory molecules in CD4^+^ and CD8^+^ T cell subsets between healthy control (HC) and moyamoya disease (MMD) groups.** Statistical significance: ns, *p* ≥ 0.05; **p* < 0.05; ***p* < 0.01; ****p* < 0.001 (Wilcoxon rank-sum test).


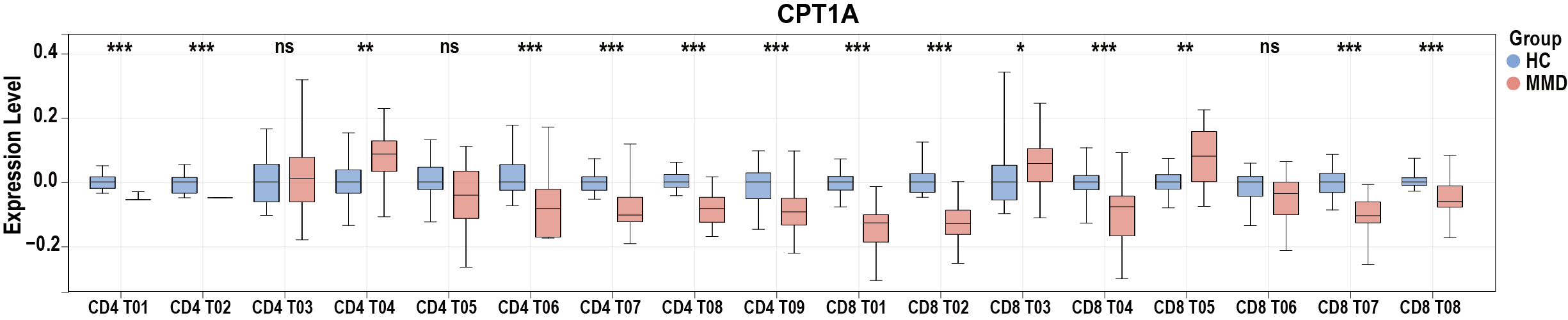


**Fig S6. Mass cytometry analysis of fatty acid metabolism–related molecules in CD4^+^ and CD8^+^ T cell subsets between healthy control (HC) and moyamoya disease (MMD) groups.** Statistical significance: ns, *p* ≥ 0.05; **p* < 0.05; ***p* < 0.01; ****p* < 0.001 (Wilcoxon rank-sum test).


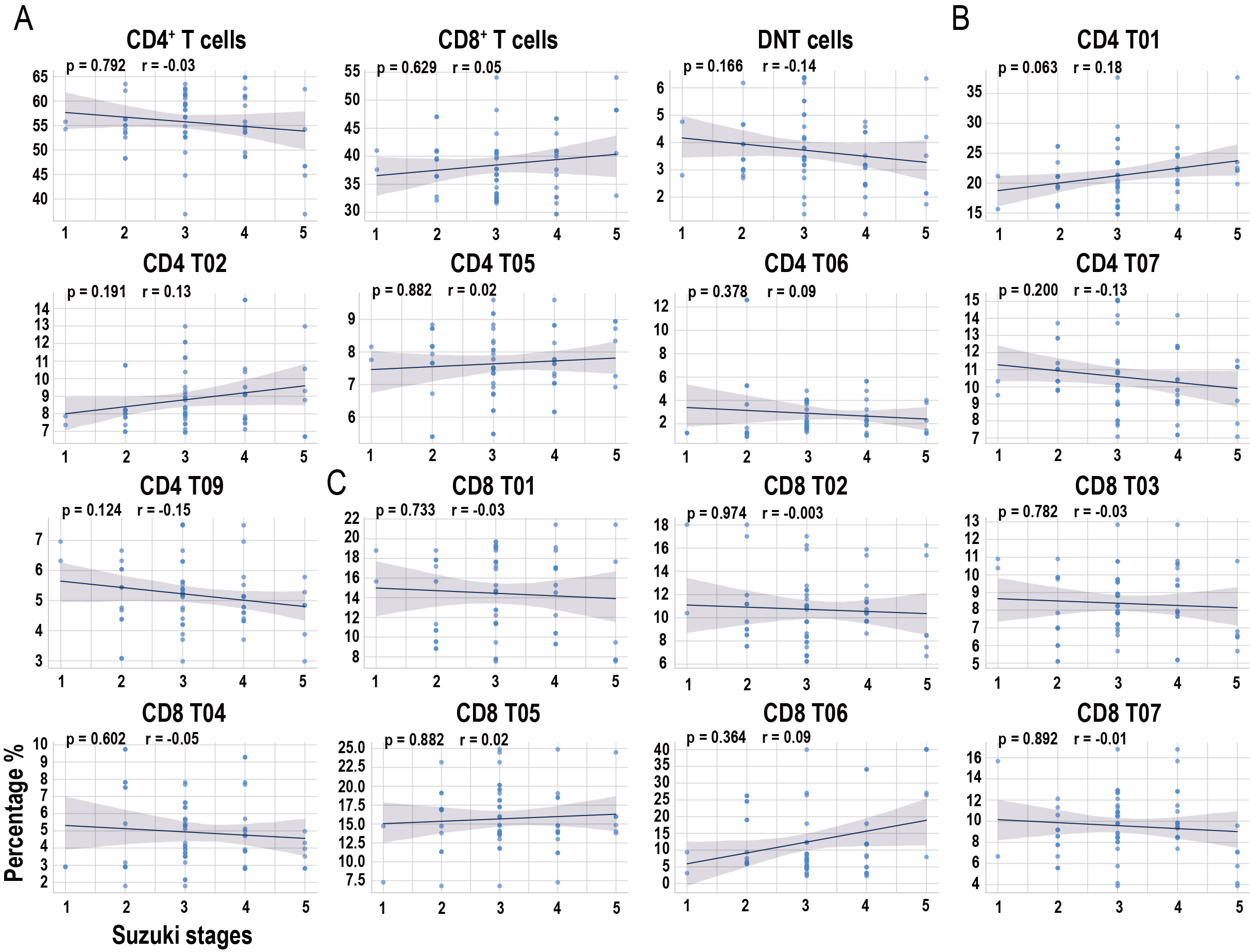


**Fig S7. Mass cytometry analysis of T cell subset proportions in relation to Moyamoya disease (MMD) progression, as defined by Suzuki stages.** (A) T cell subsets (excluding DPT cells); (B) CD4^+^ T cell subsets (excluding CD4 T03, CD4 T04, and CD4 T08); (C) CD8^+^ T cell subsets (excluding CD8 T08). *P*-values were calculated using Spearman correlation analysis.


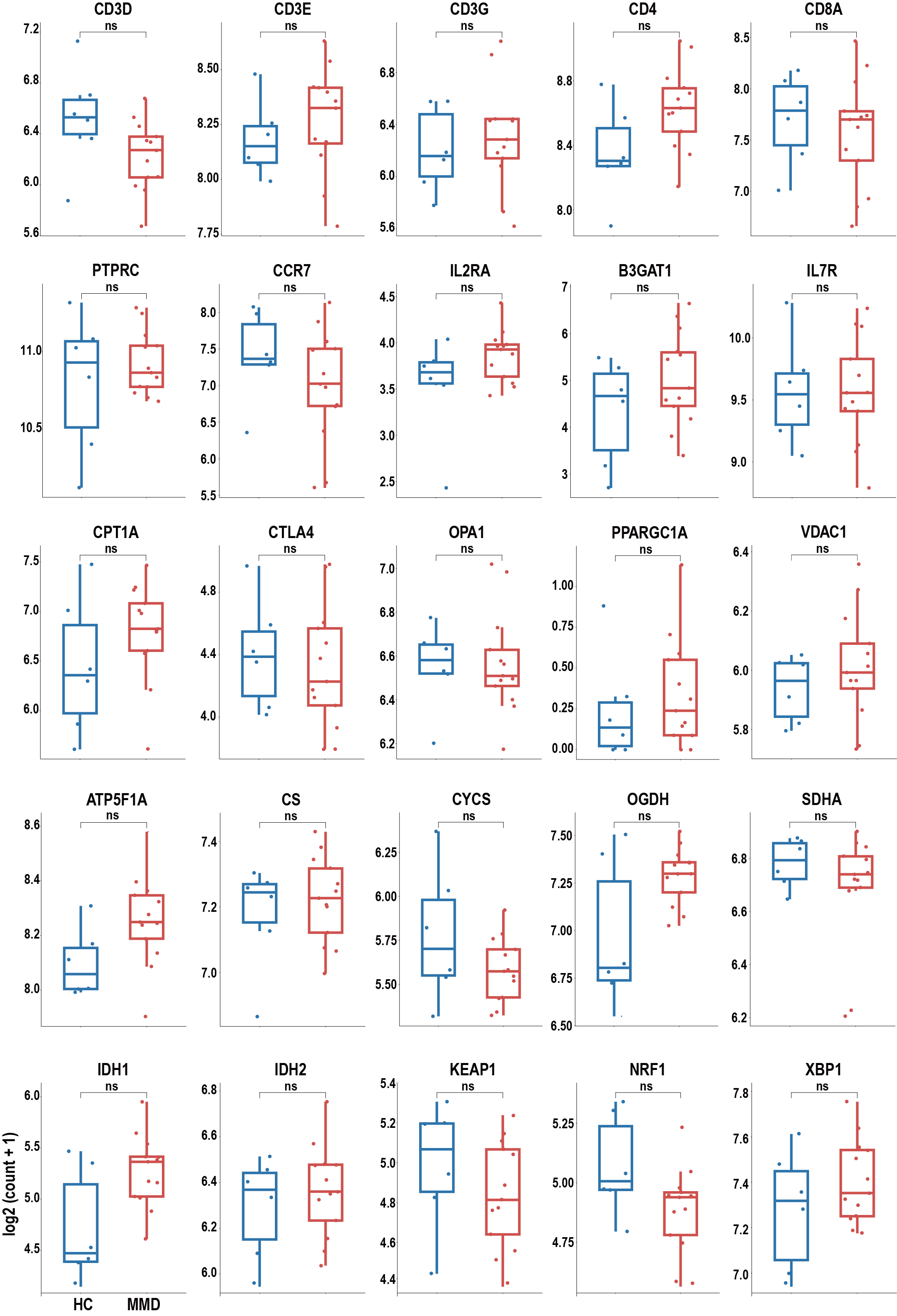


**Fig S8. Comparison of peripheral blood RNA expression levels of genes associated with mass cytometry findings between healthy control (HC) and moyamoya disease (MMD) groups.** Statistical significance: ns, *p* ≥ 0.05. Adjusted *p*-values were calculated using the Benjamini–Hochberg method implemented in the DESeq2 package in R.


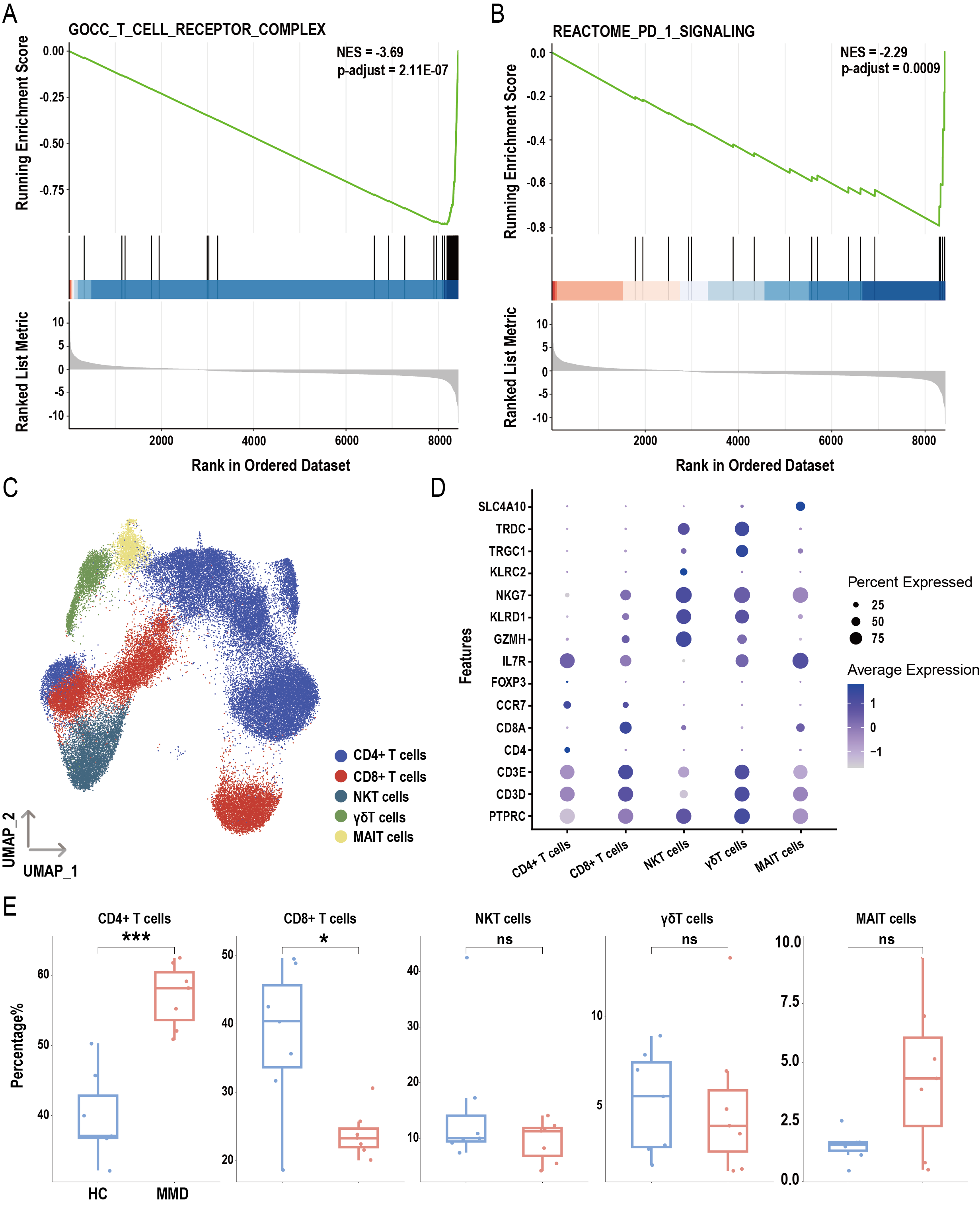


**Fig S9. Single-cell RNA-seq analysis of peripheral blood T cells.** Gene Set Enrichment Analysis (GSEA) of T cells from the moyamoya disease (MMD) group compared to the healthy control (HC) group: (A) GOCC_T_CELL_RECEPTOR_COMPLEX; (B) REACTOME_PD_1_SIGNALING. (C) UMAP plot showing dimensionality reduction and clustering of T cell subsets; (D) Heatmap illustrating the correlation between T cell subsets and cluster-specific marker genes; (E) Box plot comparing the proportions of T cell subsets between HC and MMD groups. Statistical significance: ns, *p* ≥ 0.05; **p* < 0.05; ****p* < 0.001 (Wilcoxon rank-sum test).
